# High-throughput thickness gradient screening reveals thickness and light-intensity dependent efficiency in indoor organic photovoltaics

**DOI:** 10.1039/d6ta01910b

**Published:** 2026-06-26

**Authors:** Muhammad Ahsan Saeed, Giel Swennen, Marián Prada-Cortés, Francesc Xavier Capella-Guardià, Miquel Casademont-Viñas, Xabier Rodríguez-Martínez, Jaime Martín, Koen Vandewal, Mariano Campoy-Quiles

**Affiliations:** a Institut de Ciència de Materials de Barcelona (ICMAB-CSIC) Carrer dels Til.lers 08193 Cerdanyola del Vallès Spain mcampoy@icmab.es; b Hasselt University, Institute for Materials Research (IUMAT) Martelarenlaan 42 B-3500 Hasselt Belgium; c IMEC, IUMAT Wetenschapspark 1 B-3590 Diepenbeek Belgium; d Universidade da Coruña, Centro de Investigación en Tecnoloxías Navais e Industriais (CITENI), Campus Industrial de Ferrol Campus de Esteiro S/N 15471 Ferrol Spain; e Oportunius Program, Axencia Galega de Investigación (GAIN), Xunta de Galicia Galicia Spain

## Abstract

Indoor organic photovoltaics (OPVs) are promising power sources for Internet-of-Things devices, but optimizing performance under diverse indoor lighting is challenging because the optimal thickness of the active layer depends on the competition between charge transport and recombination, as well as on the incident light spectrum in a complex manner. Here, we use a high-throughput customized blade-coating system to generate continuous active-layer thickness gradients (50–450 nm) for five binary blends comprising three wide-bandgap donors (PTQ10, PM6, D18) and three non-fullerene acceptors (o-IDFBR, eh-IDTBR, FCC-Cl). Devices were characterized under four LED spectra (2700 K, 5200 K, 6500 K, B4) using a spectrum-on-demand source. Across 600 devices, intermediate thicknesses maximize shunt resistance (*R*_P_) and fill factor, whereas thin and thick layers suffer from leakage and recombination. PTQ10:FCC-Cl shows broad thickness tolerance (≈230–410 nm) and moderate spectral stability, with PCE varying by only ≈2.6% across indoor spectra and reaching a maximum of ≈21.7% under 2700 K. Conversely, PM6:FCC-Cl attains higher PCE (≈26.4%) but is strongly thickness-sensitive, with peak efficiency realized within a narrow range (∼305 nm) and varying by ≈15% across all four spectra. Analysis of intensity- and thickness-dependent charge transport indicates that performance is governed by photon absorption, spectral overlap and insufficient *R*_P_. This work demonstrates a rapid screening method and highlights the importance of thickness- and spectrum-optimized active layers for efficient indoor OPVs.

## Abstract 2

High-throughput thickness-gradient screening reveals how active-layer thickness and indoor light spectra govern charge transport, recombination, and efficiency in indoor organic photovoltaics, identifying robust and spectrum-stable material systems.

## Graphical abstract

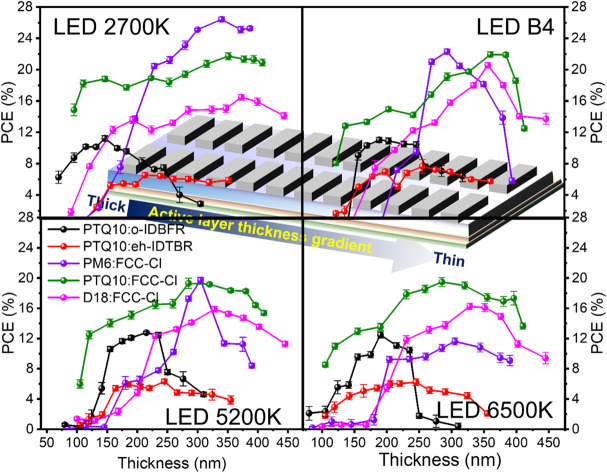

## 1 Introduction

The growing advancement of low-power indoor electronics, especially IoT devices, has created an urgent demand for efficient off-grid power sources capable of operating under dim indoor lighting conditions.^1–4^ Organic photovoltaics (OPVs) are particularly promising in this realm due to their high absorption coefficients and tunable optical properties, which allow effective energy harvesting even at low light intensities.^5–8^ Recent developments in materials and device architectures have pushed OPV power conversion efficiencies (PCEs) to nearly 36% under indoor illumination, demonstrating their potential as sustainable, maintenance-free energy solutions for applications such as powering gas sensors, wearables, and smart home technologies.^9–12^

However, despite these impressive efficiency gains, several fundamental factors influencing indoor OPV performance remain insufficiently explored. One such critical factor is the active layer thickness, which must balance enhanced photon absorption under indoor light spectra with efficient charge transport and suppressed leakage pathways. While thicker active layers can absorb more photons and reduce leakage currents, they also lead to longer charge carrier transit times, increased space-charge effects, and higher recombination losses due to the moderate carrier mobilities in organic materials.^13–15^ The wide variability in indoor illumination spectra and intensities further complicates optimization. Importantly, the impact of device thickness is strongly illumination-dependent because it modulates resistive losses differently under high- and low-light conditions.^16–19^ Under 1-sun conditions, high photocurrent densities make series resistance (*R*_S_) a dominant loss mechanism: increased thickness enlarges *R*_S_ and leads to larger voltage drops, reducing fill factor and current extraction. In contrast, under low-intensity indoor illumination, the photocurrent is orders of magnitude smaller and the voltage drop across *R*_S_ becomes negligible, while shunt resistance (*R*_P_) associated with leakage paths becomes decisive for performance, as high *R*_P_ suppresses leakage currents that strongly influence the *J*–*V* shape at low light intensity. Though thickness effects are well-studied for outdoor OPVs,^20–23^ recent efforts have begun to explore resistive losses under indoor conditions, providing a foundation for more systematic investigations. Despite these advances, several specific questions remain insufficiently addressed. In particular, it is unknown whether the optimal active-layer thickness is universal across indoor spectra or shifts with the spectral distribution of the light source, a distinction with direct practical consequences for OPV device design. Similarly, it is not well established whether a material's thickness tolerance, defined as its ability to maintain competitive PCE over a broad thickness window depends on the illumination spectrum. Furthermore, a quantitative framework that classifies indoor OPV active layers based on the trade-off between maximum efficiency and thickness tolerance is still unexplored. Addressing these questions is essential for guiding material selection and device optimization under realistic indoor operating conditions.

To effectively navigate this multidimensional optimization landscape, high-throughput screening methodologies offer a promising pathway. Techniques such as blade coating with variable speeds enable the creation of continuous thickness gradients, allowing simultaneous investigation of thickness effects on device performance under diverse indoor lighting spectra.^24–28^ This rapid, combinatorial approach accelerates the identification of optimal material blends and device architectures, surpassing the constraints of traditional trial-and-error experimentation. Moreover, they provide large self-consistent datasets that can be used for testing theoretical frameworks and/or training artificial intelligence models. Such strategies are essential to approach the Shockley–Queisser limits for indoor photovoltaics, which are estimated to be around 64% PCE for RGB light-emitting diode (LED) sources and approximately 50% for fluorescent lamps.^29,30^

**Fig. 1:**
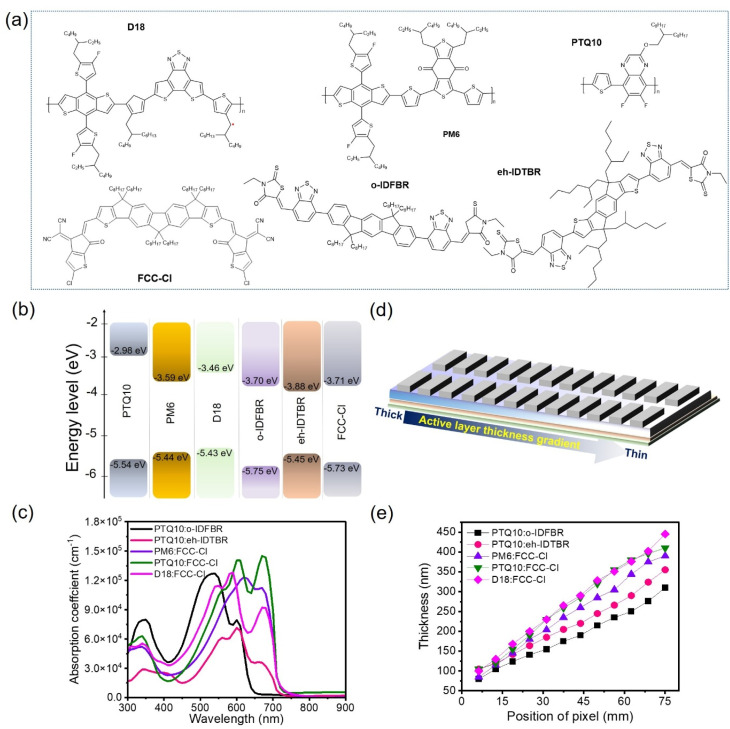
(a) Chemical structures, (b) energy levels, (c) absorption coefficients of the materials used in this study, (d) schematic illustration of 24 OPV devices with active layer thickness gradient, and (e) thickness variation of five active layers ranging from 50 to 450 nm with an estimated uncertainty of ±10 nm.

In this study, we applied our high-throughput screening approach to systematically investigate the thickness-dependent indoor performance of five active layer systems composed of binary blends of three wide-bandgap donors (PTQ10, PM6, and D18) and three wide-bandgap non-fullerene acceptors (NFAs) (o-IDFBR, eh-IDTBR, and FCC-Cl), as their chemical structures are illustrated in Fig. 1a. Using a customized blade-coating setup developed in our lab, we produced active layer thickness gradients with 12 discrete thickness values for each blend by varying the coating speed. To replicate diverse indoor environments, we characterized the devices under four different LED illumination spectra (2700 K, 5200 K, 6500 K, and B4) (corresponding to a color temperature of 5000 K, as documented in International Electrotechnical Commission (IEC))^31^ generated by our patented spectrum-on-demand light source (SOLS), resulting in a total of 2400 device measurements. The incident power and photon flux of all four indoor light sources is provided in Fig. S1. The results under indoor illumination conditions reveal notable differences: some systems exhibit pronounced thickness-dependent performance, while others show relative insensitivity. Similarly, sensitivity to illumination spectra varies, with PTQ10:FCC-Cl demonstrating remarkable stability across thickness and spectral conditions, achieving a maximum PCE of 21.7% under 2700 K LED. PM6:FCC-Cl attained the highest PCE of 26.4% under the same conditions but more thickness-dependent performance, to the best of our knowledge, the highest efficiency reported thus far for blade coated devices. Conversely, PTQ10:eh-IDTBR showed the lowest fill factor (FF) of around 35%, linked to its poor *R*_P_, but PTQ10:o-IDFBR exhibited the highest FF approaching 65% under 2700 K LED illumination. Additionally, PTQ10:o-IDFBR displayed the relatively high dependence of short-circuit current density (*J*_SC_) on illumination spectrum. These findings underscore the critical need for tailored optimization of active layer thickness and composition in response to varying indoor lighting conditions.

## 2 Experimental details

### 2.1 OPV device fabrication

Devices were fabricated in an inverted architecture with the layer sequence: glass/ITO/ZnO/active layer/MoO_3_/Ag, allowing illumination through the glass substrate. Patterned ITO substrates (100 nm thickness, 20 Ω per sq sheet resistance) were obtained from Ossila. Substrates were sequentially cleaned *via* 10 min sonication in acetone, 10% Hellmanex aqueous solution, isopropanol, and 10% NaOH solution, followed by drying with compressed air. The ZnO electron transport layer was deposited by blade-coating from an isopropanol-based nanoparticle dispersion (Avantama, N-10) and annealed at 120 °C for 10 min. Following deposition, substrates were transferred into a nitrogen-filled glovebox, where the active layers were blade-coated at varying speeds to produce a thickness gradient of approximately 50–450 nm. The hole transport layer (MoO_3_, 10 nm) and reflective Ag electrode (150 nm) were subsequently thermally evaporated (Kurt J. Lesker) through a shadow mask, defining 24 individual cells, each with an active area of 2 × 4 mm^2^.

### 2.2 Active layer materials description

Given the variety of conditions evaluated in this study, precise control over the active layer composition and fabrication is crucial. This section provides a detailed overview of the materials used and the fabrication methods employed. PTQ10, PM6, D18, FCC-Cl were purchased from Ossila and o-IDFBR and eh-IDTBR were purchased from 1-material. Complete names of the materials used are given in SI. All materials were used as received. Active layer solutions were prepared in chlorobenzene (CB, Sigma-Aldrich). Blends of PTQ10:o-IDFBR, PM6:FCC-Cl, and PTQ10:FCC-Cl were prepared at a weight ratio of 1 : 1.5, whereas PTQ10:eh-IDTBR and D18:FCC-Cl were prepared at ratios of 1 : 1 and 0.7 : 1, respectively. Solutions were stirred for 3 h at 45 °C inside a glovebox. Most active layer solutions were prepared at a concentration of 20 mg mL^−1^, regardless of the solvent; D18-based solutions were diluted to 10 mg mL^−1^ due to their high viscosity at 20 mg mL^−1^. Active layers were deposited *via* blade coating at speeds ranging from 90 mm s^−1^ to 10 mm s^−1^ across the substrate, generating a thickness gradient of approximately 450 nm to 50 nm.

### 2.3 OPV device and microstructure characterization

All samples were removed from the glovebox, and their current density–voltage (*J*–*V*) characteristics under the AM1.5G spectrum were measured using a custom-built multiplexer, which sequentially connected each cell. A XES-100S1 solar simulator (SAN-EI Electric Co., xenon arc lamp) equipped with a filter was employed to match the standard 1-sun AM1.5G spectrum. The total power output was calibrated using a silicon reference cell (Newport). For each material combination, *J*–*V* curves were recorded for 12 different active layer thicknesses, with two replicas per thickness (left and right sides), resulting in a total of 24 cells per substrate. The external quantum efficiency (EQE) of the best-performing devices for each active layer was measured at room temperature with a custom-built setup. This setup provides a broadband spectral output (UV-NIR) with the help of steady and monochromatic illumination system with Xe arc lamp (SLS401, Thorlabs). The measurement spot size was approximately 1.5 mm in diameter, smaller than the active area of the solar cells. Keysight 5500 AFM (available at ICMAB-CSIC) was used in non-contact mode to analyze the surface morphology. GIWAXS characterization was carried out at the BL11 NCD-SWEET beamline of the ALBA Synchrotron Light Source, Spain. The measurements were conducted using an X-ray energy of 12.4 keV generated through a channel-cut Si(111) monochromator, while beam focusing and collimation were achieved using Be compound refractive lenses. Diffraction patterns were collected at an incidence angle of 0.12° with an exposure time of 1 s using a Rayonix LX255-HS detector. Calibration of the scattering vector *q* was performed using a LaB_6_ standard provided by NIST, yielding a calibrated sample-to-detector distance of 199.77 mm. The in-plane (IP) and out-of-plane (OOP) linecuts were obtained after integration of the *q*_z_–*q*_r_ diffractograms as a function of the polar angle *χ*: 0–45° for OOP and 45–90° for IP linecuts, as defined in PyFAI.^32^ The diffraction peaks were fitted in Fityk using pseudo-Voigt lineshapes and a background consisting of an exponential decay function and a constant or linear baseline.^33^

### 2.4 Indoor measurement setup

For indoor light characterization, patented equipment was employed, capable of delivering a tunable spectrum that can be shaped in intensity and/or wavelength range relative to a primary light source. SOLS provides high light throughput across a broad spectral window (380–1100 nm) and offers two types of spectrally shaped output: (i) a spatially homogeneous beam with a defined spectral profile—used in this study to generate four distinct spectra for uniform areal illumination—and (ii) a spatially and spectrally split beam separating different wavelength components. The system exhibits excellent temporal intensity stability (<2%, type A solar simulator) and good spatial homogeneity in both intensity (<5%, type B) and spectral shape across the illuminated area, fulfilling IEC-recommended standards for indoor characterization. The targeted indoor illumination conditions were realized by a customized software developed within our group, which enabled the acquisition, storage, processing, and analysis of all measurement data. In the SOLS, light emitted from a xenon arc lamp is first collimated and directed to a dispersive prism, which separates the beam into its constituent wavelengths. As a result, the light distribution in the plane perpendicular to its propagation shows one axis with maximal wavelength separation and its perpendicular direction in which light (*i.e.*, each wavelength) is homogeneously distributed. A filtering mask, implemented using 3D-printed cards, is then employed to modulate the transmitted intensity at each wavelength by controlling the height of the light transmitted at each wavelength, thereby shaping the desired output spectrum. Subsequently, the spectrally separated beam is recombined with a concentrating mirror and a hexagonal light pipe. Accurate operation of the 3D-printed masking system requires calibration of the individual slits by measuring the spectrum associated with each slit to fit a mask profile for the desired spectrum. This procedure involves adjusting several parameters, including the total number of slits (40 slits are required to reproduce a complete spectrum), the integration time, and the flame scan settings. Additionally, the SOLS setup enables the change on spectra intensity without affecting the spectral shape thanks to selective light filtering before the dispersive element. Fig. S2 shows the results of indoor spectra fits performed with a set of slits measurements. Detailed descriptions of the SOLS mechanism and structure are provided in ref. 34.

## 3 Results and discussion

### 3.1 Thin-film characteristics

Fig. 1a presents the molecular structures of three wide-bandgap donor polymers—PTQ10, PM6, and D18—and three wide-bandgap NFAs—o-IDFBR, eh-IDTBR, and FCC-Cl—used in this work. The chosen materials are all wide band-gap materials, with gaps between 2.07 and 1.78 eV. PTQ10 combines a simple, high-yield synthesis with strong overlap between its absorption spectrum and common indoor light sources, making it an attractive low-cost option for indoor photovoltaics.^35,36^ D18 offers high crystallinity and efficient hole transport due to its fused-ring backbone and strong electron-withdrawing units, delivering excellent performance with Y6 or FCC-Cl.^37,38^ The acceptors eh-IDTBR and o-IDFBR were selected for their contrasting electron affinities and molecular packing behaviors, which can impact *V*_OC_ and charge mobility.^39,40^ FCC-Cl, capable of over ∼28% PCE under 2600 K LED illumination with D18 or PM6, serves as a benchmark acceptor for this study.^41^ The highest occupied molecular orbital (HOMO) and the lowest unoccupied molecular orbital (LUMO) levels shown in Fig. 1b are taken from cyclic voltammetry literature data, combined with measured ellipsometry data (optical gaps). UV-vis absorption measurements were performed to study the optical absorption properties of all binary blends. Fig. 1c shows the absorption coefficient, *α*(*λ*), for each blend from the near-UV to the near-IR region. Among these, FCC-Cl-based blends exhibit the highest *α*(*λ*), with distinct sub-peaks near 600–700 nm and a maximum value of 1.37 × 10^5^ cm^−1^ at *λ* = 675 nm for PTQ10:FCC-Cl active layer. In contrast, PTQ10:eh-IDTBR shows the lowest *α*(*λ*) of 7.78 × 10^4^ cm^−1^ at *λ* = 600 nm. PTQ10:o-IDFBR exhibits an absorption cutoff at shorter wavelengths, with a maximum value of 1.26 × 10^5^ cm^−1^ at *λ* = 530 nm. Since all spectra drop to near zero well before 800 nm, differences in the expected photocurrent are mainly attributed to the spectral breadth and peak structure within the visible range (≈520–760 nm) rather than deep-NIR harvesting. The absorption coefficients at all thickness for each blend are given in Fig. S3.

The fraction of absorbed photon flux for each blend was calculated using the transfer matrix model (TMM). The complex refractive indices (*n*, *k*) of the neat materials were taken from the literature or measured using ellipsometry.^24,42^ The *n* and *k* for the blends were constructed by linearly combining the optical constants of the corresponding neat materials, ensuring consistency with the measured absorption coefficients. Fig. S4 shows the *n* and *k* of the neat films and their corresponding blends. TMM calculations were carried out for each light source over a thickness range of 50–450 nm to evaluate thickness-dependent light harvesting. In the thin-film regime, the absorbed fraction of the incident photon flux depends in a complex manner on film thickness (Fig. S5) due to optical interference effects. As the thickness increases above ∼200 nm, the incremental gains in absorption diminished and eventually saturates over the whole spectral range of blend absorption up to a value of about 85–95%. Fig. 1d depicts the fabricated 24-device arrays arranged on a large-aspect-ratio, prepatterned ITO substrate, with equivalent left- and right-side devices. Each substrate contains 12 distinct values of the targeted parameter, with one replica per value. An active layer thickness gradient was introduced along the long axis of the substrate. This gradient was achieved *via* blade coating using modified control electronics (customized in the group) that allow precise adjustment of blade velocity and acceleration during deposition. Active layer thicknesses were measured using profilometry at each of the 12 discrete positions along the thickness gradient on the coated substrate (Fig. 1e), covering a range of approximately 50–450 nm with an estimated uncertainty of ±10 nm.

The morphological evolution is examined by the comparative grazing-incidence wide-angle X-ray scattering (GIWAXS) analysis of three active layers, namely, PTQ10:o-IDFBR, PM6:FCC-Cl, and PTQ10:FCC-Cl, which were studied as a function of film thickness (Fig. S6). The structural parameters extracted from GIWAXS measurements are summarized in Table S1 for the characteristic (100) and (010) reflections detected in each blend. In PTQ10:o-IDFBR, the (100) peak of PTQ10 locates at 2.9 nm^−1^ while no signature ascribed to o-IDFBR is observed at low *q* values. The (010) peak is fitted with a single pseudo-Voigt lineshape to represent a blend, and is located at 18.0 nm^−1^. PM6:FCC-Cl shows a much richer diffractogram, and both (100) and (010) peaks ascribed to PM6 and FCC-Cl separately are detected and fitted using pseudo-Voigt lineshapes. The (100) reflection in PM6 varies between 3.0 nm^−1^ while that of FCC-Cl does so between 3.6 nm^−1^. The (010) reflections vary between 17.2 nm^−1^ for PM6 and 18.4 nm^−1^ for FCC-Cl. In PTQ10:FCC-Cl, material-specific (100) reflections are detected, but a single (010) peak in used in this case. In PTQ10, the (100) peak is located between 2.8 nm^−1^ and that of FCC-Cl between 3.6 nm^−1^. The single (010) peak detected is located at 18.3 nm^−1^. Overall, these results confirm that peak positions, both in terms of *q* and the derived *d* values (Table S1), remain virtually constant as a function of thickness in all blends.

Notwithstanding, variations are observed essentially in the width of the peaks, therefore also in the calculated crystal coherence length *L*_c_ and the paracrystalline disorder parameter *g*. Generally, the lamellar (100) reflections ascribed to PTQ10 are broad and disordered, with *g* > 22% in all cases. In these same blends the (010) reflections are, however, much sharper and *g* decreases down to 12–13%, confirming a very low degree of paracrystalline disorder in the π-stacking direction. Actually, in PTQ10:FCC-Cl also the (100) reflection of FCC-Cl shows low *g* values of 13–15%. These values are even lower in PM6:FCC-Cl, in particular for the donor polymer (PM6) which shows *g*_100_ = 14–15.3% and *g*_010_ = 12.6–14.0%, confirming a far superior ordering than PTQ10-based blends. Also, FCC-Cl is able to preserve a good quality of order with *g*_100_ = 13.8–15.0% and *g*_010_ = 11.2–12.6% when blended with PM6. Overall, *L*_c_ and *g* data plotted as a function of thickness (Fig. S7) suggest that variations, if any, are constrained within ±20% of their average value, with PM6:FCC-Cl appearing as the most thickness-sensitive blend in terms of crystal quality (*i.e.*, *L*_c_ and *g*). Actually, only in such a blend *L*_c_ and *g* tend to increase and decrease, respectively, as the thickness increases, thus indicating improved ordering in thicker films.

We have also evaluated the integrated peak area from the corresponding linecuts after normalization by the film thickness (Fig. S7). As no change of film orientation is observed as a function of film thickness (Fig. S6), the peak areas should be a good approximation to the actual volume of diffracting crystallites in the films. It is therefore observed that in PTQ10:o-IDFBR blends, the peak area of both (100) and (010) reflections of PTQ10 maximize at *ca.* 150–200 nm, thus correlating with an increase in the parallel resistance observed in devices. In PM6:FCC-Cl, peak areas show a similar yet less pronounced pattern with the area maximizing at film thicknesses between 200–300 nm, a range which also agrees with the improved parallel resistance observed in devices. Finally, in PTQ10:FCC-Cl it is observed how the crystallinity of PTQ10 increases steadily up to 350 nm approximately, correlating with the roll-off in performance observed for thicker films from that threshold thickness (350 nm) upwards. There is, thus, a plausible correlation between the observed improvement of the parallel resistance in devices and the increase in the diffraction peak areas, hence in the amount of crystalline material in the active layer (and in particular of the donor polymer).

Moreover, atomic force microscopy (AFM) was performed to examine the thickness-dependent surface morphology of the three active-layer systems. The corresponding height and phase images (Fig. S8) reveal differences in surface roughness (*R*_q_) and phase contrast across the various film thicknesses. Among the studied blends, PM6:FCC-Cl consistently exhibits higher roughness values, whereas the PTQ10-based systems maintain comparatively smoother and more uniform surfaces over a broad thickness range. Notably, PTQ10:FCC-Cl displays the smoothest morphology at intermediate thicknesses (100–175 nm), with roughness values below 10 nm and relatively uniform phase characteristics. Although surface roughness slightly increases for thicker films (280–340 nm), the morphological evolution remains more gradual than in the other systems. This enhanced morphological stability is consistent with the broad thickness tolerance observed for PTQ10-based active layers.

### 3.2 PV performance under 1-sun illumination

**Fig. 2:**
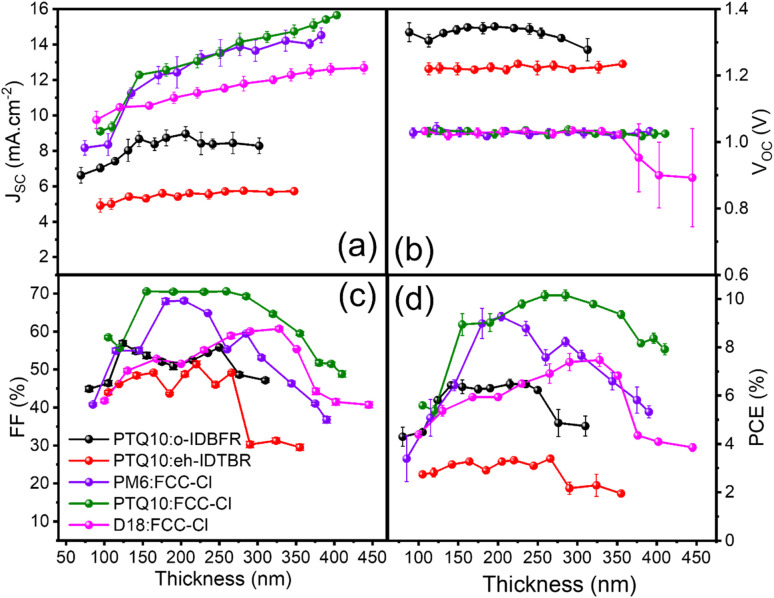
Photovoltaic parameters under 1-sun illumination, (a) *J*_SC_, (b) *V*_OC_, (c) FF, and (d) PCE, plotted against active layer thickness gradient.

Fig. 2 presents the variation of photovoltaic parameters as a function of active-layer thickness for the five binary systems under 1-sun illumination. For each blend and illumination condition, measurements were performed on two independent substrates fabricated under identical conditions, with 2 replica devices per thickness value per substrate, giving a total of *n* = 4 devices per thickness value. All reported photovoltaic parameters represent the mean of these four measurements, and error bars represent the standard deviation. Among them, FCC-Cl-based devices exhibited the most pronounced increase in *J*_SC_ with increasing thickness (Fig. 2a). This behavior can be attributed to the stronger absorption coefficient and broader spectral coverage of FCC-Cl, which enhance photon harvesting as the optical path length increases. In contrast, the other active layers showed smaller *J*_SC_ gains with thickness, likely due to less efficient light absorption in the longer-wavelength region and increased carrier recombination at larger thicknesses. The open-circuit voltage (*V*_OC_) remained largely stable across thickness variations for most active layers, indicating minimal thickness-induced changes in the effective bandgap or interfacial energetics (Fig. 2b). An exception was observed for D18:FCC-Cl devices, where *V*_OC_ decreased at higher thicknesses which might be associated with increased energetic disorder when charge transport becomes diffusion-limited in thicker films.^41^ FF was highest (∼70%) and notably stable in PTQ10:FCC-Cl devices, reflecting a balanced charge transport and low series resistance even at increased thickness (Fig. 2c). Other blends demonstrated stronger FF sensitivity to thickness, particularly PM6:FCC-Cl, where FF losses were likely driven by a mismatch in electron and hole mobilities and a higher probability of bimolecular recombination in thicker films. The corresponding PCE trends are shown in Fig. 2d. PTQ10:FCC-Cl devices achieved the highest PCE of 10.2%, followed by PM6:FCC-Cl (9.39%), D18:FCC-Cl (7.46%), PTQ10:o-IDFBR (6.58%), and PTQ10:eh-IDTBR (3.36%). A summary of photovoltaic parameters under 1-sun illumination conditions is given in Table 1.

**Table 1:** A summary of photovoltaic parameters under 1-sun illumination

	Thickness (nm)	*V*_OC_ (V)	*J*_SC_ (mA cm^−2^)	*J*_EQE_ (mA cm^−2^)	FF (%)	PCE (%)
PTQ10:o-IDFBR	235	1.36	8.87	8.33	54.51	6.58
PTQ10:eh-IDTBR	220	1.21	5.56	5.29	49.27	3.36
PM6:FCC-Cl	205	1.03	13.16	12.5	68.64	9.39
PTQ10:FCC-Cl	285	1.03	14.19	13.9	69.12	10.2
D18:FCC-Cl	290	1.04	11.96	11.4	60.01	7.46

**Fig. 3:**
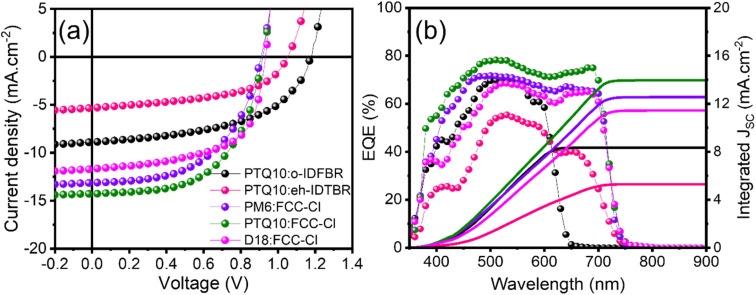
(a) *J*–*V* characteristic curves under 1-sun illumination, and (b) EQE spectra of best performing cells for each active layer.

Fig. 3a presents the *J*–*V* characteristics of the highest-performing devices for each active-layer system under 1-sun, representing the optimal performance achieved in this study. These curves clearly illustrate the differences in *J*_SC_ and *V*_OC_ among the blends. Fig. 3b displays the corresponding EQE spectra. All active layers exhibit broad photoresponse in their respective absorption regions, and the integrated photocurrent densities (*J*_EQE_) calculated from the EQE spectra are in close agreement with the *J*_SC_ values obtained from the *J*–*V* measurements, confirming the reliability of the photovoltaic characterization. Notably, PTQ10:FCC-Cl devices exhibited the strongest photoresponse, with EQE values approaching 80% and broad spectral coverage extending well into the near-infrared region. FCC-Cl-based blends in general maintained high EQE between 600–800 nm, consistent with their strong light absorption in this range. In contrast, PTQ10:o-IDFBR devices showed an EQE response with cutoff before 700 nm, reflecting their narrower absorption window. PTQ10:eh-IDTBR devices exhibited both a suppressed response in the 350–450 nm region and a generally lower EQE across the full spectral range compared to the other active layers, consistent with their reduced measured *J*_SC_ and total PCE.

### 3.3 PV performance under indoor illumination

**Fig. 4:**
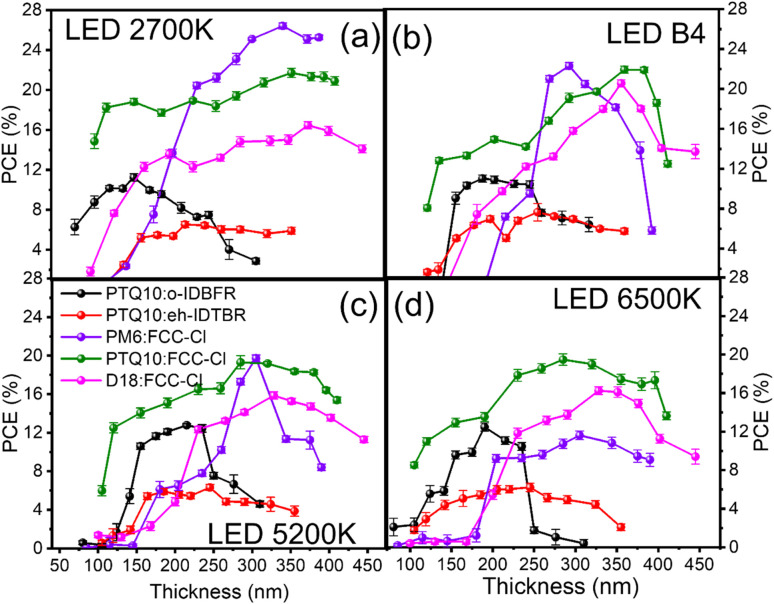
PCE plotted against active layer thickness gradient through high-throughput experimentation under (a) LED 2700 K, (b) LED B4, (c) LED 5200 K, and (d) LED 6500 K.

Interestingly, some of the active layers showed pronounced thickness and spectrum dependencies, which triggered our interest to explore further examination under varied thickness and illumination conditions. Under 2700 K LED illumination, thickness strongly regulates the PCE of all active layers and pronounced in FCC-Cl blends (Fig. 4a). PM6:FCC-Cl and PTQ10:FCC-Cl sustain >20% over broad ranges, peaking at 26.4% (∼344 nm) and 21.7% (∼355 nm), respectively, consistent with strong spectral overlap with the warm source and tolerable transport/recombination losses even in thick films. D18:FCC-Cl generally improves to 16.5% with thickness (∼376 nm) before a mild rollover at the thickest films. In contrast, the non-FCC-Cl blends peak at much thinner layers—PTQ10:o-IDFBR at 11.24% (∼155 nm) and PTQ10:eh-IDTBR at 6.5% (∼220 nm)—and decline thereafter, indicating limited ability to leverage additional thickness (transport constraints). Largely, the warm spectrum favors red-absorbing FCC-Cl blends that tolerate, or prefer, thick-film architectures, whereas non-FCC-Cl blends require thinner, tightly optimized layers. Under B4 LED illumination (Fig. 4b), performance trends differ notably from the 2700 K case, with reduced tolerance to very thick films in most blends. PTQ10:o-IDFBR shows a steady increase in efficiency from negligible values at thin layers to a maximum of 11.1% (∼175 nm), followed by a steep drop beyond 200 nm. PTQ10:eh-IDTBR peaks at 7.6% (∼245 nm) and remains in the 6–7% range, thereafter, suggesting early saturation of absorption. PM6:FCC-Cl delivers a high 22.3% (∼285 nm) but exhibits pronounced thickness sensitivity, with substantial losses at both thin and very thick layers. PTQ10:FCC-Cl maintains strong performance across a broad window, peaking at 22.0% (∼355 nm) and declining only slightly at the thickest films. D18:FCC-Cl improves steadily to 20.6% (∼351 nm) before a moderate drop. Generally, B4 LED conditions favor intermediate-to-thick films in FCC-Cl blends, but optimal ranges are narrower compared with 2700 K, and thickness-induced losses are more severe in PM6:FCC-Cl and PTQ10:o-IDFBR. Under 5200 K LED illumination (cooler light source), most blends exhibit moderate efficiencies with less pronounced benefits from extreme thicknesses (Fig. 4c). PTQ10:o-IDFBR rises steadily to 12.7% (∼215 nm) before declining, while PTQ10:eh-IDTBR peaks modestly at 6.3% (∼245 nm) and drops thereafter, indicating limited spectral overlap with 5200 K LED. PM6:FCC-Cl shows a late surge, reaching 19.8% (∼305 nm), but suffers steep losses beyond 340 nm, reflecting transport limitations under these conditions. PTQ10:FCC-Cl maintains high and stable output, peaking at 19.3% (∼285 nm) with minimal drop at greater thickness, whereas D18:FCC-Cl improves to 15.9% (∼328 nm) before tapering off. Overall, cooler white illumination reduces the relative advantage of thick films compared with warmer spectra, with FCC-Cl blends still outperforming others but showing narrower optimal thickness windows.

Under 6500 K LED illumination (Fig. 4d), performance is generally lower for non-FCC-Cl active layers and more thickness-sensitive. PTQ10:o-IDFBR reaches its maximum of 12.4% (∼190 nm) before a sharp drop, falling below 2% at the thickest films, reflecting strong recombination penalties under blue-rich spectra. PTQ10:eh-IDTBR peaks modestly at 6.2% (∼245 nm) and remains relatively stable within the mid-thickness range. PM6:FCC-Cl shows limited benefit from increasing thickness, achieving a modest maximum of 11.7% (∼305 nm), suggesting reduced spectral overlap compared with warmer sources. PTQ10:FCC-Cl remains the most robust performer, peaking at 19.4% (∼285 nm) and maintaining high efficiency across much of the tested range. D18:FCC-Cl improves steadily to 16.3% (∼328 nm) before declining at greater thickness. Mostly, blue-rich illumination diminishes the gains from thicker films, with FCC-Cl blends—especially PTQ10:FCC-Cl—retaining the highest efficiencies and stability. Across all light sources, FCC-Cl-based active layers consistently outperform their non-FCC-Cl counterpart, though the magnitude and thickness dependence vary with spectral characteristics. Warm illumination (2700 K) yields the highest absolute PCEs, particularly for PM6:FCC-Cl and PTQ10:FCC-Cl, which sustain >20% over broad thickness ranges due to strong red-near-IR absorption and efficient transport in thick films. At intermediate spectra (B4 LED), high PCEs persist but with narrower optimal thickness windows, especially in PM6:FCC-Cl and PTQ10:o-IDFBR, where performance drops sharply outside the peak range. While, under cooler light sources (5200 K and 6500 K), the advantage of thick films diminishes, and non-FCC-Cl blends show pronounced sensitivity to thickness, often peaking at mid-range values before rapid decline. PTQ10:FCC-Cl emerges as the most thickness-tolerant and spectrally robust active layer, maintaining high efficiency across all conditions, whereas PTQ10:o-IDFBR and PTQ10:eh-IDTBR require thinner, tightly optimized films for competitive output. Importantly, direct comparison of the PCE-thickness trends under 1-sun illumination (Fig. 2d) and the four LED spectra (Fig. 4) reveals that the optimal active-layer thickness is strongly dependent on illumination intensity and, to a lesser extent, on spectral distribution. Under 1-sun conditions, the PCE-thickness relationship is mainly governed by broadband absorption and charge-transport/recombination trade-offs, resulting in material-specific optima with a pronounced decline at large thickness. In contrast, under LED illumination, both the magnitude and the shape of the PCE-thickness dependence change substantially. Even for the same active layer, the thickness yielding maximum PCE under 1-sun does not coincide with that under LED conditions, and the tolerance to thickness variations either narrows or broadens depending on the light source. These results demonstrate that changing the illumination environment fundamentally reshapes the thickness-performance relationship in OPVs, signifying the necessity of optimizing active-layer thickness under the intended operating illumination. Table 2 summarizes the indoor photovoltaic performance of best-performing devices based on blade coated active layers. For comparison, Table S2 summarizes the state-of-the-art indoor OPV values, mainly from spin coated devices.

**Table 2:** A summary of photovoltaic parameters under four indoor light sources

	Thickness (nm)	*J*_EQE_ (µA cm^−2^)	*J*_SC_ (µA cm^−2^)	*V*_OC_ (mV)	FF (%)	PCE (%)	*P*_out_ (µW cm^−2^)
**LED 2700 K (*P***_**in**_ **: 952 µW cm** ^**−2**^ **)**							
PTQ10:o-IDFBR	155	215	222	743	64.8	11.2	106.6
PTQ10:eh-IDTBR	220	171	178	987	35.4	6.6	62.3
PM6:FCC-Cl	345	422	461	939	58.1	26.4	251.3
PTQ10:FCC-Cl	355	394	414	953	52.6	21.7	206.5
D18:FCC-Cl	375	316	327	969	49.4	16.5	157.1

**LED B4 (*P***_**in**_ **: 494 µW cm** ^**−2**^ **)**							
PTQ10:o-IDFBR	175	127	154	733	48.3	11.2	55.3
PTQ10:eh-IDTBR	245	122	131	831	34.6	7.6	37.5
PM6:FCC-Cl	285	202	210	906	58.1	22.3	110.1
PTQ10:FCC-Cl	355	187	202	940	57.1	22.0	108.6
D18:FCC-Cl	350	157	179	984	57.8	20.6	101.7

**LED 5200 K (*P***_**in**_ **: 364 µW cm** ^**−2**^ **)**							
PTQ10:o-IDFBR	215	95	113	769	53.2	12.69	45.8
PTQ10:eh-IDTBR	245	78	80.1	831	34.6	6.3	23.0
PM6:FCC-Cl	305	133	129	880	63.3	19.8	72.0
PTQ10:FCC-Cl	320	122	134	936	56.1	19.3	70.2
D18:FCC-Cl	330	102	104	961	57.7	15.9	57.9

**LED 6500 K (*P***_**in**_ **: 411 µW cm** ^**−2**^ **)**							
PTQ10:o-IDFBR	190	114	135	769	39.1	9.9	40.5
PTQ10:eh-IDTBR	245	81	82.92	856	36.04	6.2	25.6
PM6:FCC-Cl	305	157	150	804	39.7	11.7	48.1
PTQ10:FCC-Cl	285	144	151	939	56.3	19.4	79.3
D18:FCC-Cl	330	120	122	962	57.0	16.3	66.7

### 3.4 Intensity-thickness-dependent device performance

**Fig. 5:**
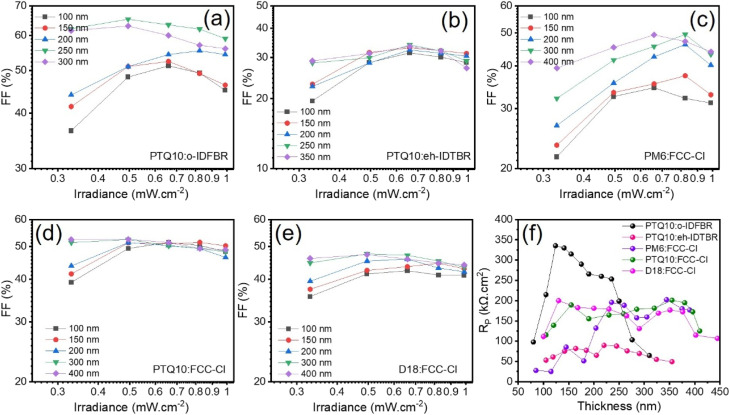
FF dependence on low-intensity irradiance for (a) PTQ10:o-IDFBR, (b) PTQ10:eh-IDTBR, (c) PM6:FCC-Cl, (d) PTQ10:FCC-Cl, (e) D18:FCC-Cl, and (f) thickness-dependent *R*_P_ for each active layer.

Typically, series resistance (*R*_S_), which is originated from the electrodes, transport, and bulk active layers, plays a decisive role in regulating the PCE of OPVs under standard 1-sun illumination.^43^ This *R*_S_ can lower both FF and *J*_SC_ in thick active layers due to limited charge mobility of organic semiconductors. The impact of resistive elements on the indoor performance of OPVs are often examined by using equivalent circuit model.^44,45^ For indoor illumination conditions where *J*_SC_ decreases dramatically, often by two to three orders of magnitude, the impact of *R*_S_ on the voltage drop (*J*_*R*_S__) becomes marginal. Instead, the reduced operating voltage in such low-intensity environments makes the impact of dark-measured *R*_P_, associated with leakage current, more pronounced. Both *R*_S_ and *R*_P_ depend strongly on the active-layer thickness. Thinner layers tend to reduce resistive losses, though shunt pathways may vary due to imperfections within the film. Comparing devices of different thicknesses therefore offers a practical approach to assess performance under weak illumination. Fig. 5a–e shows the variation of FF with varied light intensity on log scale for five active layers across thickness gradient (100–450 nm). FF tends to increase with thickness up to an optimal range, depending on the material. At high intensities, FF is primarily limited by *J*_*R*_S__ drop and more pronounced in o-IDFBR-based devices. While, in low-light region, thinner films exhibit lowest FF for each active layer. PM6:FCC-Cl and PTQ10:FCC-Cl maintained the highest FF of over 50% at optimal thicknesses (250–300 nm).

Since indoor FF is strongly influenced by the *R*_P_, the *R*_P_ values for each active layer across thickness gradient are also provided in Fig. 5f. For PTQ10:o-IDFBR, *R*_P_ rises sharply from ∼98 kΩ cm^2^ at 80 nm to ∼336 kΩ cm^2^ at 124 nm, indicating reduced shunt pathways, then decreases to ∼64 kΩ cm^2^ at 310 nm, suggesting large leakage current (corresponds to reduced FF) at higher thicknesses. PTQ10:eh-IDTBR shows a similar trend with lower absolute resistances, peaking at ∼82 kΩ cm^2^ at 164 nm and declining to ∼49 kΩ cm^2^ at 355 nm. PM6:FCC-Cl behaves irregularly, with *R*_P_ rising from ∼28 kΩ cm^2^ at 85 nm to ∼196 kΩ cm^2^ at 235 nm, then moderately decreasing to ∼177 kΩ cm^2^ at 390 nm, reflecting thickness-dependent uniformity and defect formation. PTQ10:FCC-Cl shows an increase up to ∼201 kΩ cm^2^ at 355 nm, followed by a slight decline (∼125 kΩ cm^2^ at 410 nm), indicating initial suppression of shunt paths with eventual microstructural defects. D18:FCC-Cl exhibits higher *R*_P_ values, peaking near 199 kΩ cm^2^ at 168–200 nm, and gradually decreasing to ∼107 kΩ cm^2^ at 445 nm, suggesting moderate thicknesses optimally suppress leakage while very thick layers introduce recombination pathways. Intermediate thicknesses generally maximize *R*_P_, minimizing shunt-related losses, whereas very thin layers lack coverage and very thick layers develop defects or inhomogeneities. FF benefits from high *R*_P_, which suppresses leakage current, but at very large thicknesses, lower *R*_P_ can limit FF even when absorption is adequate. The distribution of FF against *R*_P_ for each active layer under all indoor light sources is given in Fig. S9. Apparently, PTQ10:FCC-Cl and PTQ10:eh-IDTBR demonstrated the most spectrum-insensitive behavior among all active layers.

**Fig. 6:**
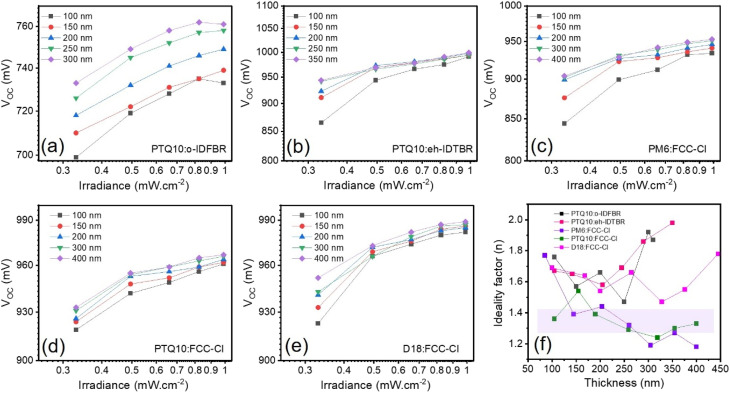
*V*_OC_ dependence on low-intensity irradiance for (a) PTQ10:o-IDFBR, (b) PTQ10:eh-IDTBR, (c) PM6:FCC-Cl, (d) PTQ10:FCC-Cl, (e) D18:FCC-Cl, and (f) ideality factor (*n*).

To understand how device performance evolves under varying illumination, we used the concept of critical resistance (*R*_PC_ = FF·*V*_OC_/*J*_SC_).^46^ This represents the minimum *R*_P_ required for the entire current to pass through the device. Geometrically, *R*_PC_, defines the slope of the line connecting *J*_SC_ and *V*_OC_. When the actual *R*_P_ falls below *R*_PC_, the device operates under conditions that can significantly reduce performance at the given light intensity. Fig. 6a–e shows the variation in *V*_OC_ against light intensity for each active layer across the thickness gradient. Since *J*_SC_ ∝ photon flux (*φ*) and *V*_OC_ ∝ ln(*φ*) + constant, *R*_PC_ increases with decreasing light intensity. Thin active layers, which typically have lower *R*_P_, reach *R*_PC_ at higher light intensities, resulting in an early linear decline of *V*_OC_ with reduced intensity. After securing sufficiently large *R*_P_ (>100 kΩ cm^2^), the performance is limited by the *V*_OC_ drop with decreasing light intensity. In this region, the high ideality factors (as in the case of o-IDFBR) have tremendously negative impact on the performance, reflecting a sharp decline in *V*_OC_. The thickness-dependent ideality factors for each active layer are shown in Fig. 6f. Relatively high ideality factors of active layers due to dominance of trap-assisted recombination contributes to surge in *V*_OC_ loss with reduced intensity even at low thickness values, as in the case of PM6:FCC-Cl.

The thickness and illumination dependence of *J*_SC_ highlights distinct behaviors among the active layers, reflecting differences in absorption and charge transport (Fig. S10). For all active layers, *J*_SC_ generally increases with thickness, but the rate of growth, saturation thickness, and absolute current levels vary. PTQ10:o-IDFBR exhibits moderate thickness sensitivity: under red-rich 2700 K and B4 LEDs, *J*_SC_ increases gradually from ∼200–104 µA cm^−2^ at ∼85–105 nm to maxima of ∼256 µA cm^−2^ and ∼185 µA cm^−2^ at the thickest films (∼250–300 nm), while blue-rich LED sources (5200 K and 6500 K) deliver lower currents (∼117–148 µA cm^−2^) that saturate around 230–250 nm. This indicates that red-NIR absorption dominates photocurrent generation, whereas limited photon flux in the blue region constrains performance in thinner layers. While, PTQ10:FCC-Cl shows broad thickness tolerance with steady *J*_SC_ growth across all sources. Under 2700 K LED, currents rise from 344 µA cm^−2^ at 105 nm to a maximum of 419 µA cm^−2^ at 410 nm, plateauing beyond ∼320 nm. B4 LED produces similar monotonic increases, reaching 208 µA cm^−2^, whereas cooler LED sources (5200 K, 6500 K) yield lower maxima (∼145–163 µA cm^−2^) but maintain a similar thickness dependence. The high absolute currents under warm spectra reflect strong overlap with the blend's red-NIR absorption, allowing thicker films to benefit from increased optical path length without significant recombination losses. In contrast, PM6:FCC-Cl is highly thickness- and illumination-dependent: under LED 2700 K LED, *J*_SC_ rises steeply from 22 µA cm^−2^ at 85 nm to 489 µA cm^−2^ at 390 nm, while under B4 LED it saturates around 224 µA cm^−2^, and under blue-enhanced LEDs (5200 K and 6500 K) *J*_SC_ reaches ∼130–155 µA cm^−2^ quickly, demonstrating that high-energy photons are efficiently absorbed even in thin films. On a whole, PTQ10:FCC-Cl is the most stable blend with respect to thickness variations and shows moderate light-source sensitivity, PTQ10:o-IDFBR is more illumination-dependent with moderate thickness sensitivity, and PM6:FCC-Cl is the most thickness-sensitive with narrower optimal thickness ranges under different spectra. The measured *J*_SC_ values where compared with the maximum expected photocurrent, calculated from the absorbed photon flux using TMM. Overall, simulated *J*_SC_ shows reasonable agreement with the experimental data, taken into account the error on the estimation of *n* and *k* values for the blends from the neat material values. For PTQ10:eh-IDFBR, PM6:FCC-Cl and D18:FCC-Cl, the measured *J*_SC_ is strongly reduced as compared to the calculated one, which we attribute to an insufficiently large *R*_P_ for thicknesses below 150–200 nm. The absence of strong interference effects in the experimental *J*_SC_ is attributed to the fact that over each pixel, the thickness varies by about 9 nm due to the gradient. These results emphasize that optimizing both active layer thickness and material selection according to the spectral environment is crucial for maximizing indoor photovoltaic performance. The *J*_SC_ calculated from the EQE spectra was found to be consistent with the measured *J*_SC_ under all indoor light sources (Fig. S11). It was measured for each active layer using incident photon flux and EQE spectra of the best-performing devices. The dependence of charge recombination on active layer thickness was evaluated through both *J*_SC_*versus* light intensity slopes (Fig. S12) and the *n* values extracted from *V*_OC_*versus* light intensity measurements on log–log scale, as mentioned in Fig. 6f. Across all five active layers, slopes remained close to unity at thinner films, indicating minimal bimolecular recombination under low-intensity indoor illumination. As thickness increased, subtle reductions in slope were observed for PTQ10:o-IDFBR and PTQ10:eh-IDTBR, suggesting a slight increase in trap-assisted recombination, which is consistent with their relatively high *n* values (1.58–1.87 and 1.58–1.98, respectively). This indicates that thicker layers in these blends start to experience enhanced recombination *via* traps, possibly due to longer charge transport pathways or increased disorder. In contrast, PM6:FCC-Cl exhibited consistently high slopes (0.914–0.960) and relatively lower *n* values (1.19–1.44), implying efficient charge extraction and limited trap-assisted recombination even at higher thicknesses. Similarly, PTQ10:FCC-Cl maintained slopes close to unity (0.939–0.988) with moderately low *n* (1.24–1.54), reflecting broad thickness tolerance and robust charge collection with minimal thickness-induced recombination. D18:FCC-Cl showed slightly larger *n* values (1.47–1.78) and moderate slopes (0.900–0.960), indicating that thicker films are increasingly prone to trap-assisted recombination, although whole performance remains satisfactory. Collectively, these trends reveal that thickness strongly influences recombination pathways: blends like PM6:FCC-Cl and PTQ10:FCC-Cl are more resilient to thickness variations, maintaining near-ideal charge extraction, whereas PTQ10:o-IDFBR, PTQ10:eh-IDTBR, and D18:FCC-Cl show enhanced trap-assisted recombination at higher thicknesses. These observations correlate well with *J*_SC_ behavior, where thicker films of the latter blends exhibit reduced photocurrent gains under low-intensity indoor lighting, highlighting the interaction between absorption enhancement and recombination penalties. Across all layers, LED 2700 K consistently generates the highest charge, due to better spectral overlap with the red/NIR absorption of the materials and a higher photon flux in the 500–700 nm region, followed by LED B4, with LED 5200 K and 6500 K yielding a lower absorbed photon flux.

However, across all four LED sources, the absolute variations in device performance due to spectral distribution are smaller than those caused by changes in light intensity. FCC-Cl-based active layers perform slightly better under 2700 K LED illumination, but the relative differences in PV parameters remain modest compared with the large intensity-driven changes. While o-IDFBR and eh-IDTBR blends exhibit some spectral sensitivity, the dominant factors limiting performance are recombination and leakage at low light intensities rather than the spectrum itself. These observations highlight that, for indoor OPVs, careful consideration of light intensity is far more critical than spectral distribution.

## 4 Conclusions

We systematically explored the thickness- and spectrum-dependent performance of five binary OPV blends across a wide range of illumination conditions using high-throughput blade-coated thickness gradients (50–450 nm). Under 1-sun illumination, FCC-Cl-based devices exhibited the strongest photocurrent enhancement with increasing thickness, with PTQ10:FCC-Cl achieving the highest PCE of 10.2% and a stable FF (∼70%), while PM6:FCC-Cl and D18:FCC-Cl were more thickness-sensitive due to transport and recombination limitations. Under indoor lighting, warm 2700 K LEDs favored thicker FCC-Cl blends, with PM6:FCC-Cl reaching 26.4% PCE at ∼344 nm and PTQ10:FCC-Cl achieving 21.7% at ∼355 nm. Cooler spectra (5200–6500 K) reduced the advantage of thick films, particularly for non-FCC-Cl blends, which peaked at 6–12% PCE in thinner layers. Intermediate thicknesses (∼200–350 nm for FCC-Cl; ∼150–250 nm for non-FCC-Cl) generally optimized *R*_P_ and FF, while extreme thicknesses were prone to trap-assisted recombination. Also, TMM calculations validated a strong dependence of the maximum achievable photocurrent on active-layer thickness and illumination spectrum, with rapid increases in *J*_SC_ at low thicknesses followed by saturation and interference effects in thicker films. These results endorse that achieving high-performance indoor OPV requires careful balancing of optical absorption, charge transport, and recombination pathways, tailored to the spectral characteristics of the illumination. Within the typical range of indoor LED illumination, variations in total incident power appear to have a more pronounced impact on device performance than the specific spectral distribution, particularly for systems with broad spectral robustness like PTQ10:FCC-Cl. The observed differences among blends highlight the importance of designing material systems with both spectral compatibility and thickness tolerance for low-intensity energy applications.

## Author contributions

MAS: conceptualization, formal analysis, methodology, investigation, writing – original draft, writing – review and editing. GS: visualization, investigation. MCV: methodology, visualization, investigation. FXCG: investigation, resources, sample preparation, formal analysis. KV: resources, formal analysis, writing – review and editing. MPC, XRM & JM: GIWAXS measurements and analysis. MCQ: conceptualization, supervision, project administration, validation, funding acquisition, writing – review and editing.

## Conflicts of interest

The authors have no conflicts to disclose.

## Supplementary Material

TA-014-D6TA01910B-s001

## Data Availability

The data that support the findings of this study have been included as part of the supplementary information (SI). Supplementary information: the full name of the active layer materials, further details on the illumination condictions and spectra, absorption data, ellipsometrically deduced refractive index and extinction coefficient for single and blend materials, as well as additional device data. See DOI: https://doi.org/10.1039/d6ta01910b.
