# Supplementary material for: High-throughput thickness gradient screening reveals thickness and light-intensity dependent efficiency in indoor organic photovoltaics
Source: J Mater Chem A Mater. 2026 Jun 26. Online ahead of print. doi: 10.1039/d6ta01910b (PMC13338873; doi:10.1039/d6ta01910b)
Supplement: TA-OLF-D6TA01910B-s001 [file TA-OLF-D6TA01910B-s001.pdf]

Supplementary Information (SI) for Journal of Materials Chemistry A.
This journal is © The Royal Society of Chemistry 2026

Supporting Information

High-throughput thickness gradient screening reveals thickness and light-intensity dependent efficiency in indoor organic photovoltaics

Muhammad Ahsan Saeed [1], Giel Swennen [2,3], Marián Prada-Cortés[4], Francesc Xavier Capella-Guardià [1], Miquel Casademont-Viñas [1], Xabier Rodríguez-Martínez [4], Jaime Martín[4,5], Koen Vandewal [2,3], Mariano Campoy-Quiles [1,*]

[1] Institute of Materials Science of Barcelona (ICMAB-CSIC), Campus UAB, Bellaterra 08193, Spain

[2] Hasselt University, Institute for Materials Research (IUMAT), Martelarenlaan 42, B-3500 Hasselt, Belgium

[3] imec, IUMAT, Wetenschapspark 1, B-3590 Diepenbeek, Belgium

[4] Universidade da Coruña, Centro de Investigación en Tecnoloxías Navais e Industriais (CITENI), Campus Industrial de Ferrol, Campus de Esteiro S/N, 15471 Ferrol, Spain

[5] Oportunius Program, Axencia Galega de Investigación (GAIN), Xunta de Galicia, Galicia, Spain

[*]Corresponding author: mcampoy@icmab.es

Active layer materials names

PTQ10: Poly[(thiophene)-alt-(6,7-difluoro-2-(2- hexyldecyloxy)quinoxaline)]

PM6: Poly[(2,6-(4,8-bis(5-(2-ethylhexyl-3-fluoro)thiophen-2-yl)-benzo[1,2-b:4,5-b']dithiophene))-alt-(5,5-

(1',3'-di-2-thienyl-5',7'-bis(2-ethylhexyl)benzo[1',2'-c:4',5'-c']dithiophene-4,8-dione)]

D18: Poly[(2,6-(4,8-bis(5-(2-ethylhexyl-3-fluoro)thiophen-2-yl)-benzo[1,2-b:4,5-b']dithiophene))-alt-5,5'-

(5,8-bis(4-(2-butyloctyl)thiophen-2-yl)dithieno[3',2':3,4;2'',3'':5,6]benzo[1,2-c][1,2,5]thiadiazole)]

o-IDFBR: (5Z,5'Z)-5,5'-((7,7'-(6,6,12,12-Tetraoctyl-6,12-dihydroindeno[1,2-b]fluorene-2,8-

diyl)bis(benzo[c][1,2,5]thiadiazole-7,4-diyl))bis(methanylylidene))bis(3-ethyl-2-thioxothiazolidin-4-one)

eh-IDTBR: (5Z)-3-Ethyl-2-sulfanylidene-5- [[4-[9,9,18,18-tetrakis(2-ethylhexyl)-15-[7-[(Z)-(3-ethyl-4-oxo-2-sulfanylidene-1,3-thiazolidin-5-ylidene)methyl]-2,1,3-benzothiadiazol-4-yl]-5,14-dithiapentacyclo[10.6.0.03,10.04,8.013,17]octadeca-1(12),2,4(8),6,10,13(17),15-heptaen-6-yl]-2,1,3-benzothiadiazol-7-yl]methylidene]-1,3-thiazolidin-4-one

FCC-Cl: 2-(2-chloro-6-oxo-5,6-dihydro-4Hcyclopenta[b]thiophen-4-ylidene)-malononitrile

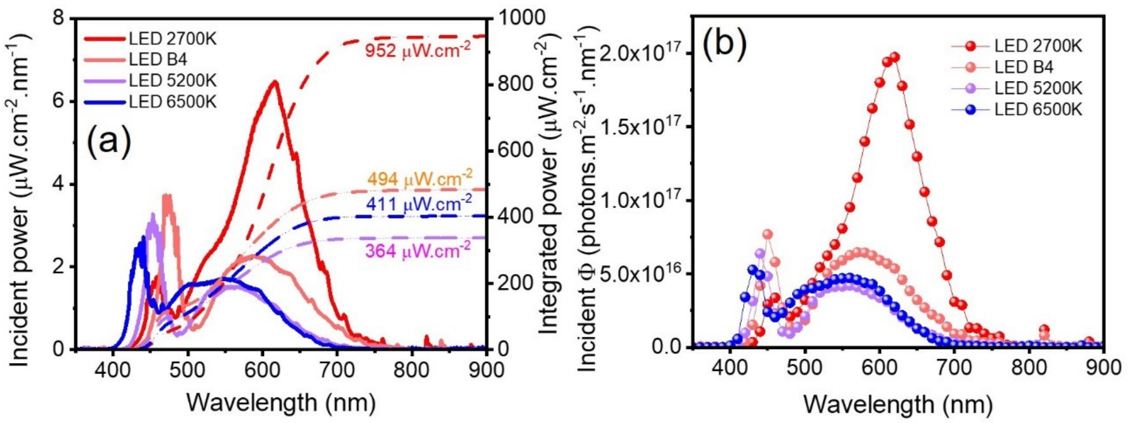

Figure S1. (a) The irradiation power spectra and integrated power densities curves of the indoor light sources and (b) The incident photon flux of indoor light sources.

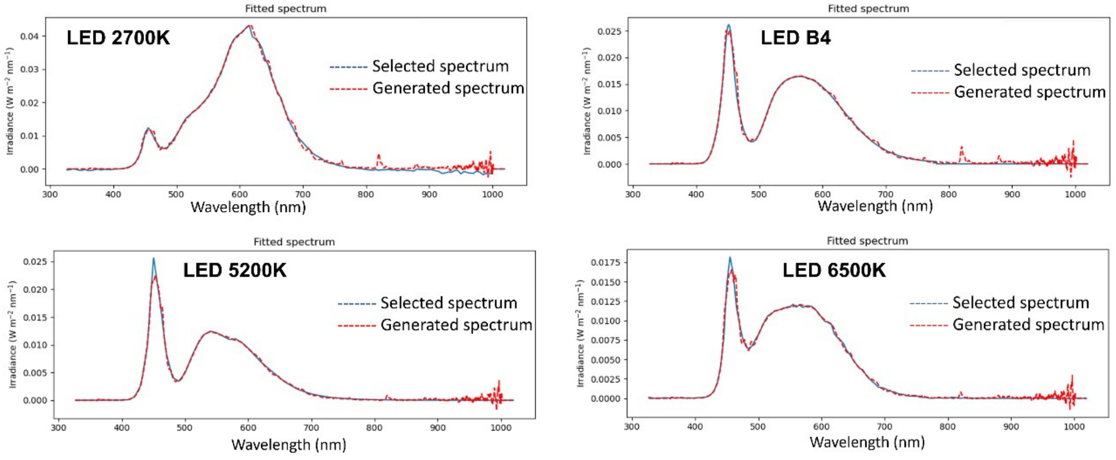

Figure S2. SOLS generated and fitted light-illumination spectra for all four LED light sources.

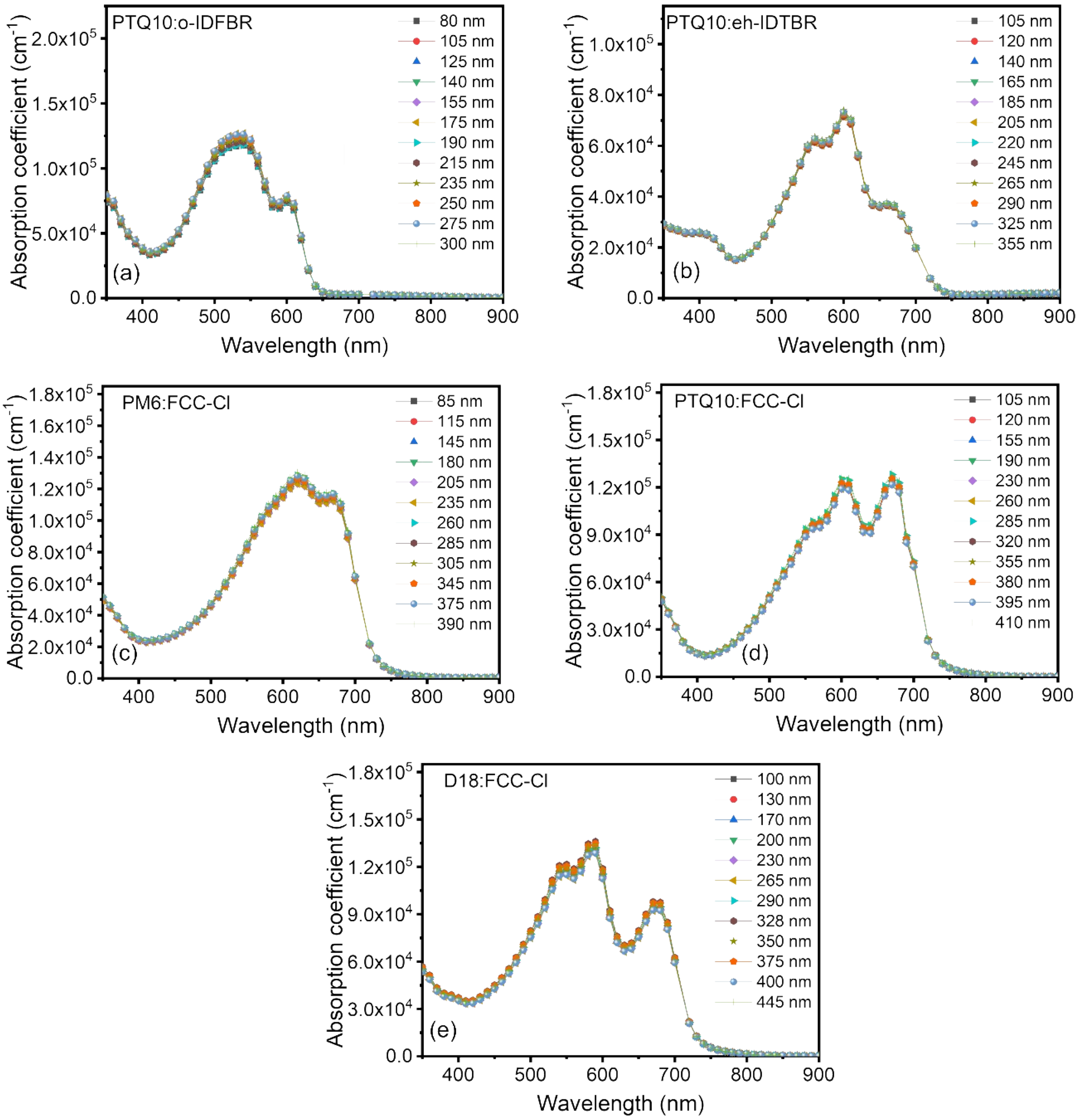

Figure S3. Absorption coefficient of active layers against varied thickness. (a) PTQ10:o-IDFBR, (b) PTQ10:eh-IDTBR, (c) PM6:FCC-Cl, (d) PTQ10:FCC-Cl, and (e) D18:FCC-Cl.

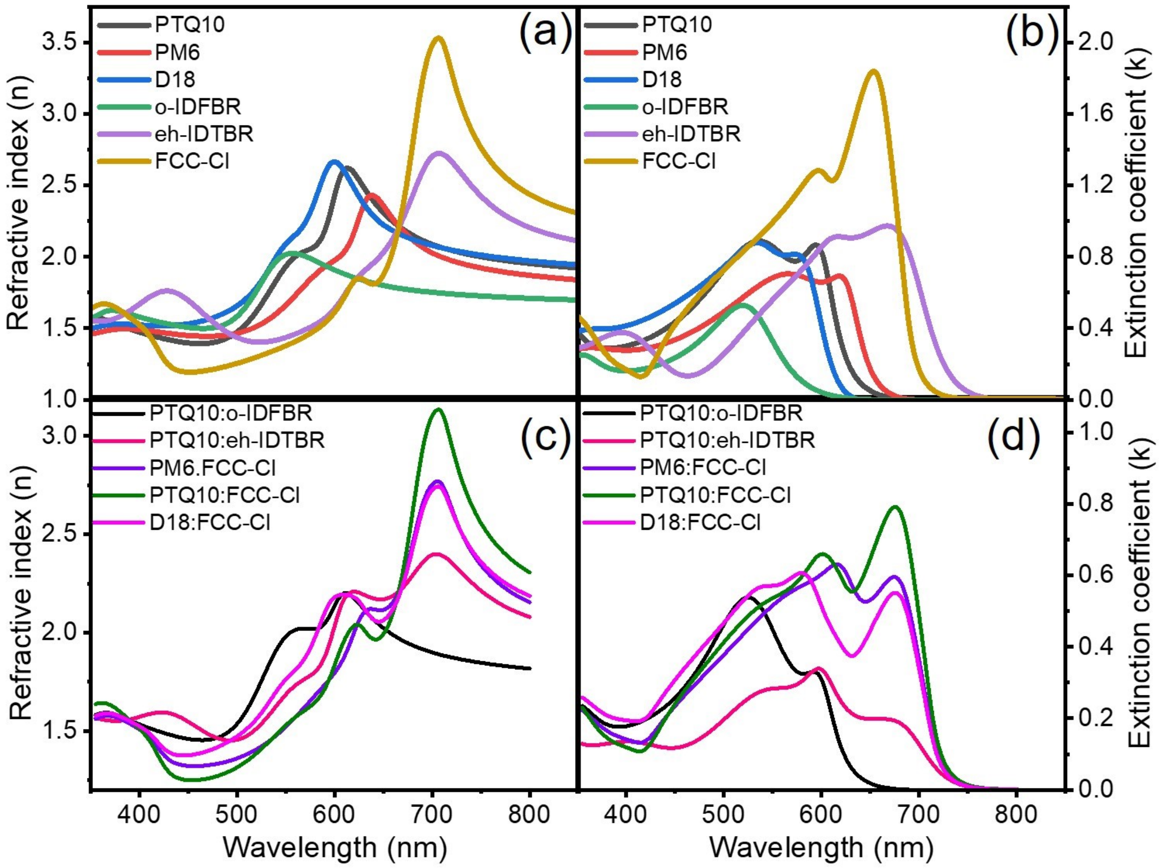

Figure S4. Refractive index (n) (a-c) and extinction coefficient (k) (b-d) of individual materials and the binary blend, respectively.

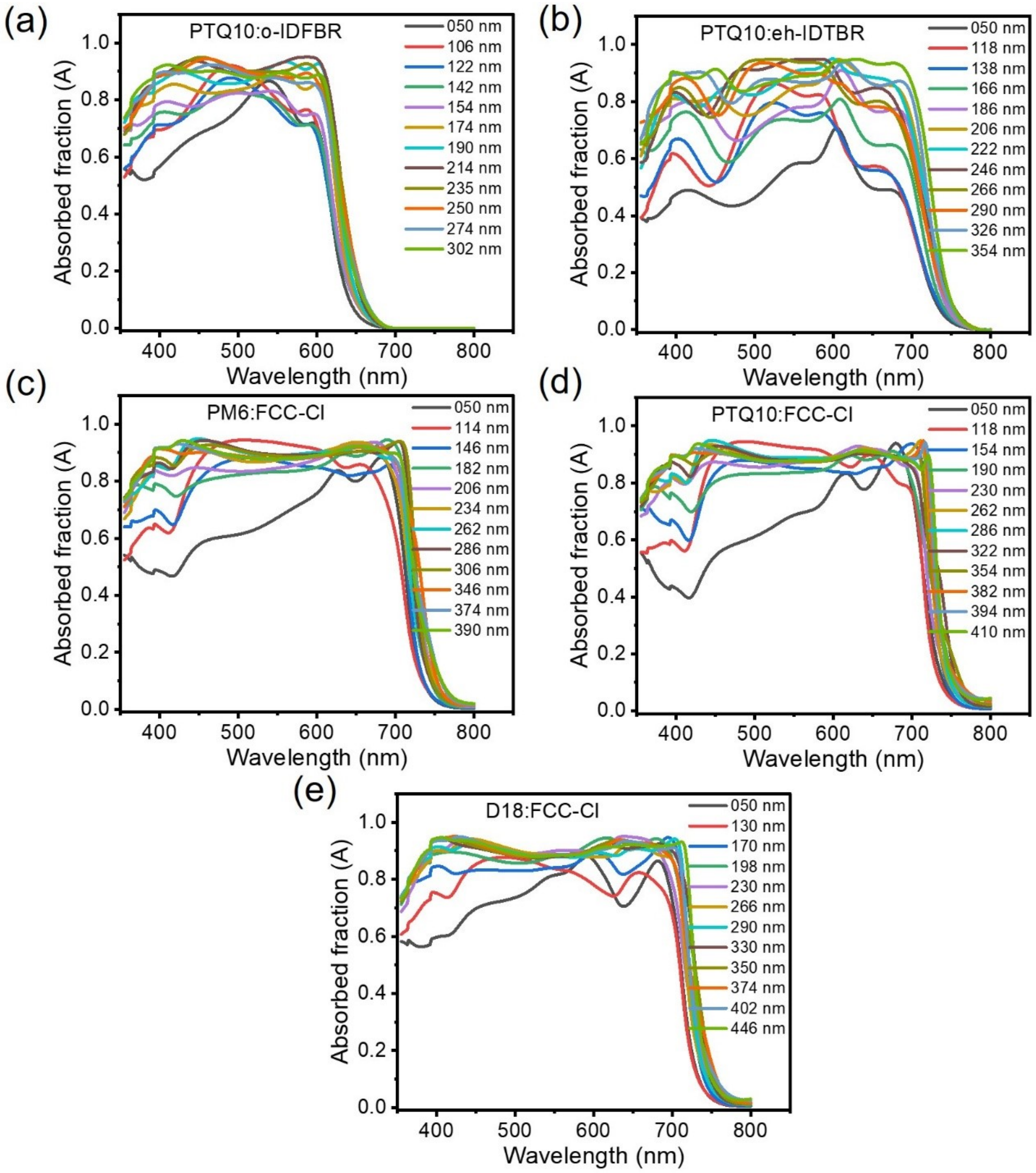

Figure S5. Absorbed fraction of active layers was calculated through transfer matrix model and plotted against active layer thickness gradient. (a) PTQ10:o-IDFBR, (b) PTQ10:eh-IDTBR, (c) PM6:FCC-Cl, (d) PTQ10:FCC-Cl, and (e) D18:FCC-Cl.

GIWAXS analysis

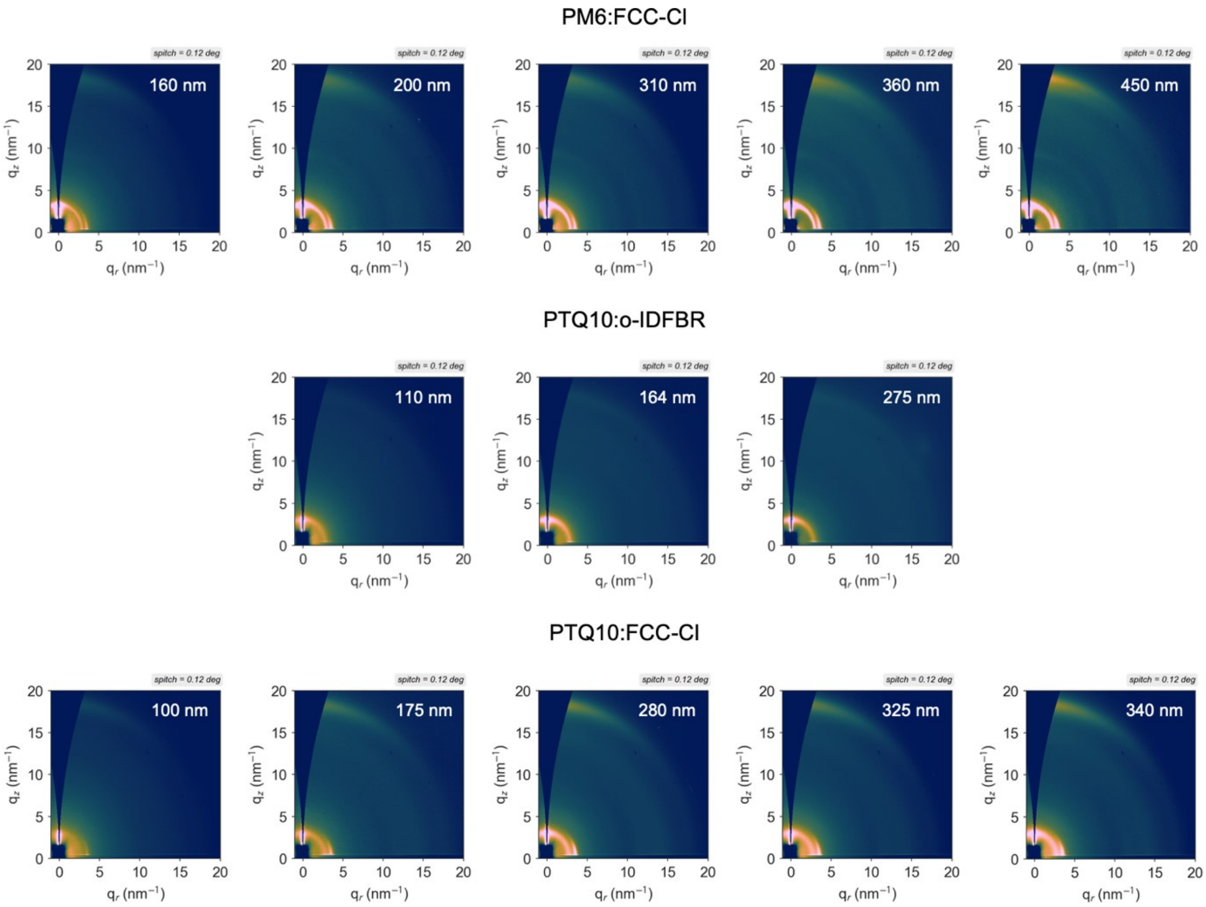

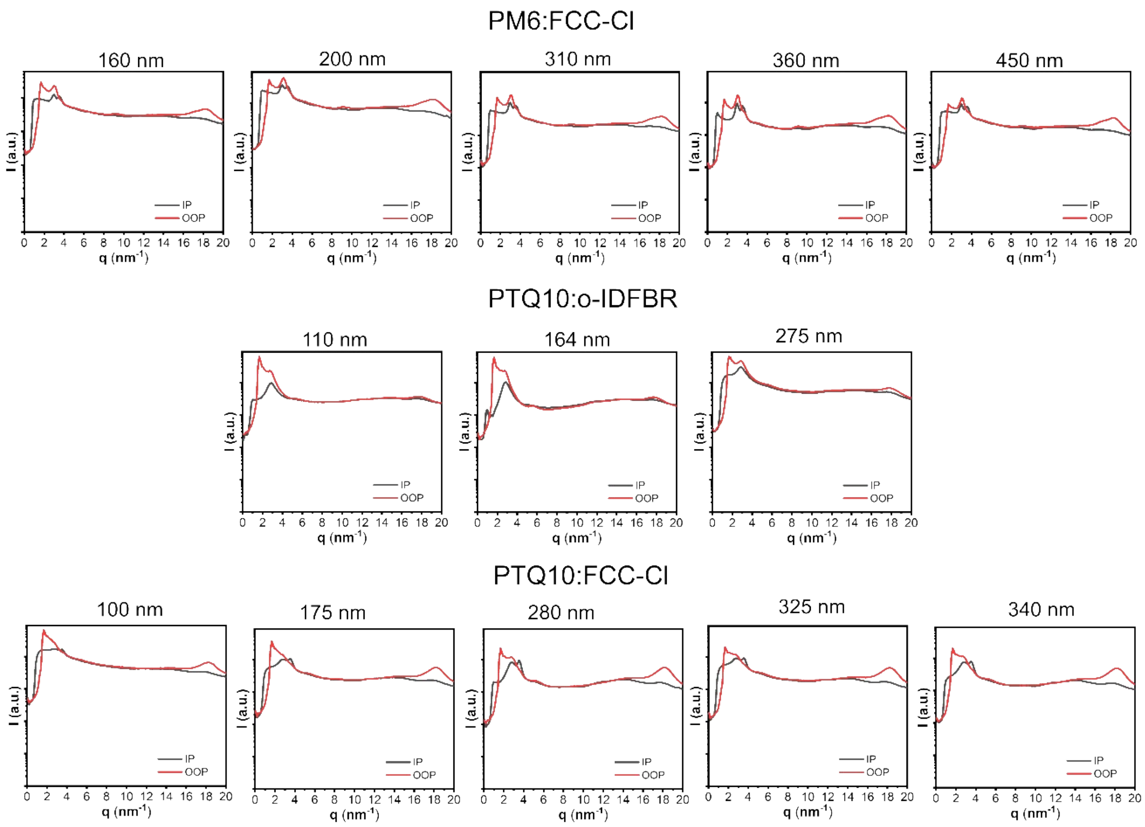

Figure S6. 2D GIWAXS patterns of some of the blends studied in this work (PM6:FCC-Cl, PTQ10:o-IDFBR, PTQ10:FCC-Cl) as a function of film thickness (indicated in the corresponding images). The 2D GIWAXS patterns were measured at an incident angle of 0.12°.

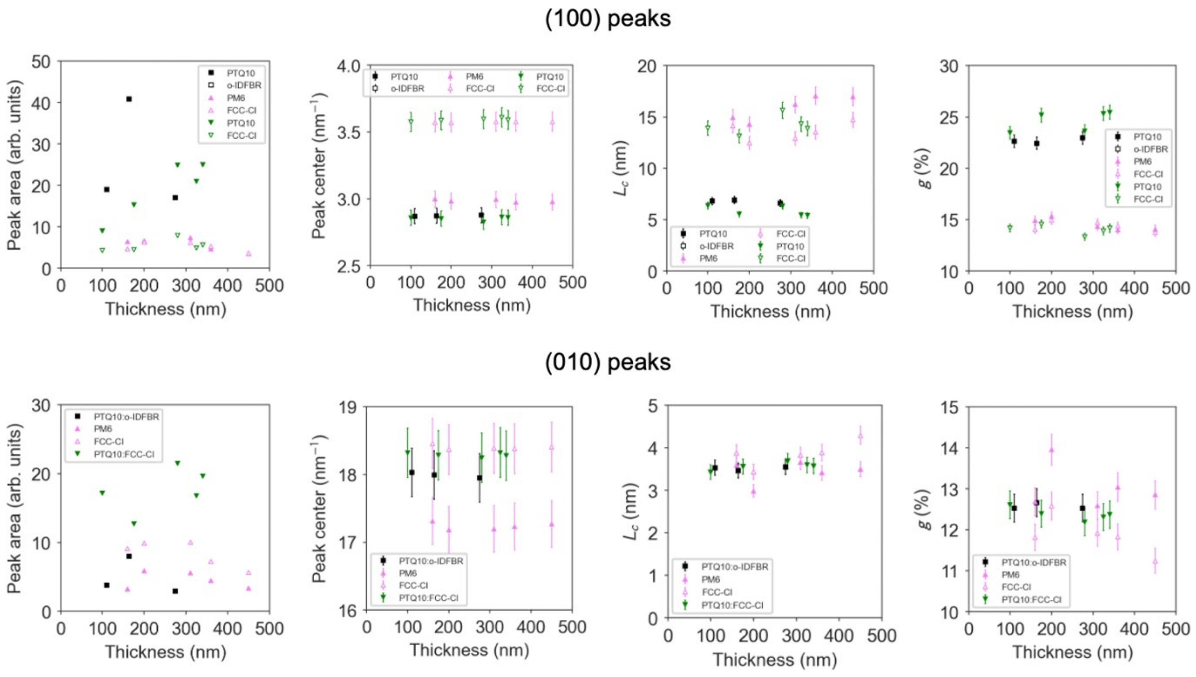

Figure S7. Peak parameters as a function of thickness obtained after fitting the corresponding IP and OOP linecuts of the 2D GIWAXS patterns acquired at an incident angle of 0.12º. From the IP linecuts, the (100) peak parameters were extracted. Note that o-IDFBR did not show any characteristic (100) diffraction peak (cf. FCC-Cl). From the OOP linecuts, the (010) peak parameters were extracted. In this case, a single (010) peak was used in fitting the PTQ10:o-IDFBR and PTQ10:FCC-Cl linecuts, while in the case of PM6:FCC-Cl two distinct peaks were detected, here ascribed to PM6 and FCC-Cl separately.

Table S1. Extracted parameters of active layers' blend from GIWAXS measurements.

			**PTQ10:o-IDFBR**				
**Thickness (nm)**	**Material**	**MI (hkl)**	**q (nm^{-1})**	**HWHM**	**d (nm)**	**L_C (nm)**	**g (%)**
110	PTQ10	100	2.87	0.46	2.19	6.8	22.6
	Blend	010	18.0	0.89	0.35	3.5	12.5
165	PTQ10	100	2.87	0.46	2.19	6.9	22.4
	Blend	010	18.0	0.91	0.35	3.5	12.7
275	PTQ10	100	2.88	0.48	2.18	6.6	22.9
	Blend	010	17.95	0.89	0.35	3.5	12.5
			**PM6:FCC-Cl**				
160	PM6	100	2,998	0,21	2,10	15,0	14,9
	FCC-Cl	100	3,572	0,222	1,76	14,2	14,1
	PM6	010	17,32	0,876	0,36	3,6	12,7
	FCC-Cl	010	18,46	0,81	0,34	3,9	11,8
200	PM6	100	2,982	0,22	2,11	14,3	15,3
	FCC-Cl	100	3,572	0,252	1,76	12,5	15,0
	PM6	010	17,19	1,052	0,37	3,0	14,0
	FCC-Cl	010	18,37	0,914	0,34	3,4	12,6
310	PM6	100	2,995	0,194	2,10	16,2	14,4
	FCC-Cl	100	3,576	0,243	1,76	12,9	14,7
	PM6	010	17,2	0,857	0,37	3,7	12,6
	FCC-Cl	010	18,39	0,822	0,34	3,8	11,9
360	PM6	100	2,975	0,184	2,11	17,1	14,0
	FCC-Cl	100	3,577	0,232	1,76	13,5	14,4
	PM6	010	17,23	0,921	0,36	3,4	13,0
	FCC-Cl	010	18,38	0,808	0,34	3,9	11,8
450	PM6	100	2,976	0,185	2,11	17,0	14,1
	FCC-Cl	100	3,578	0,213	1,76	14,7	13,8
	PM6	010	17,27	0,897	0,36	3,5	12,9
	FCC-Cl	010	18,41	0,731	0,34	4,3	11,2
			**PTQ10:FCC-Cl**				
100	PTQ10	100	2,86	0,49	2,20	6,4	23,5
	FCC-Cl	100	3,58	0,23	1,76	13,9	14,2
	Blend	010	18,32	0,92	0,34	3,4	12,6
	PTQ10	100	2,85	0,57	2,20	5,5	25,2

	FCC-Cl	100	3,59	0,24	1,75	13,1	14,6
	Blend	010	18,29	0,88	0,34	3,6	12,4
280	PTQ10	100	2,83	0,50	2,22	6,3	23,6
	FCC-Cl	100	3,60	0,20	1,75	15,7	13,3
	Blend	010	18,25	0,85	0,34	3,7	12,2
325	PTQ10	100	2,86	0,58	2,20	5,4	25,3
	FCC-Cl	100	3,61	0,22	1,74	14,3	13,9
	Blend	010	18,32	0,87	0,34	3,6	12,3
340	PTQ10	100	2,86	0,58	2,20	5,4	25,5
	FCC-Cl	100	3,59	0,23	1,75	13,9	14,2
	Blend	010	18,28	0,88	0,34	3,6	12,4

The crystal coherence length (L_C) was calculated using the Scherrer equation, L_C = 2πK/FWHM, where K is the shape factor = 1.0 and FWHM is full width half maximum (cf. HWHM, half width half maximum). The intermolecular distance (d) was calculated by 2π/q where q is the scattering vector. The structural disorder within the paracrystalline lattice was quantified using the paracrystalline disorder parameter (*g*) derived from the first-order diffraction peaks, where a smaller g value corresponds to a more ordered lattice arrangement.

$$g\ (\%) = \sqrt{\frac{FWHM}{2\pi q}}$$

AFM analysis

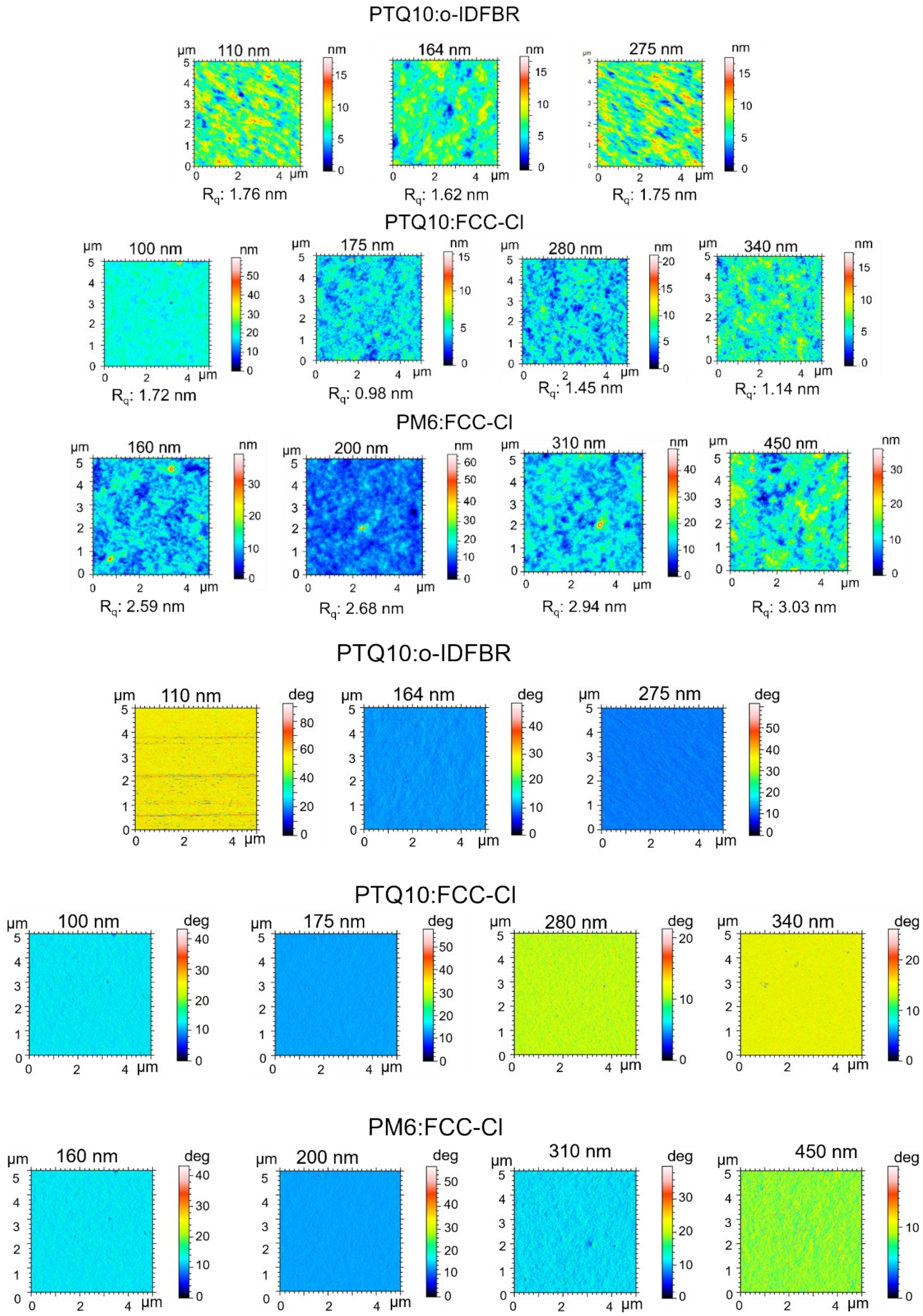

Figure S8. AFM height and phase images of PTQ10:o-IDFBR, PTQ10:FCC-Cl, and PM6:FCC-Cl with variable thickness.

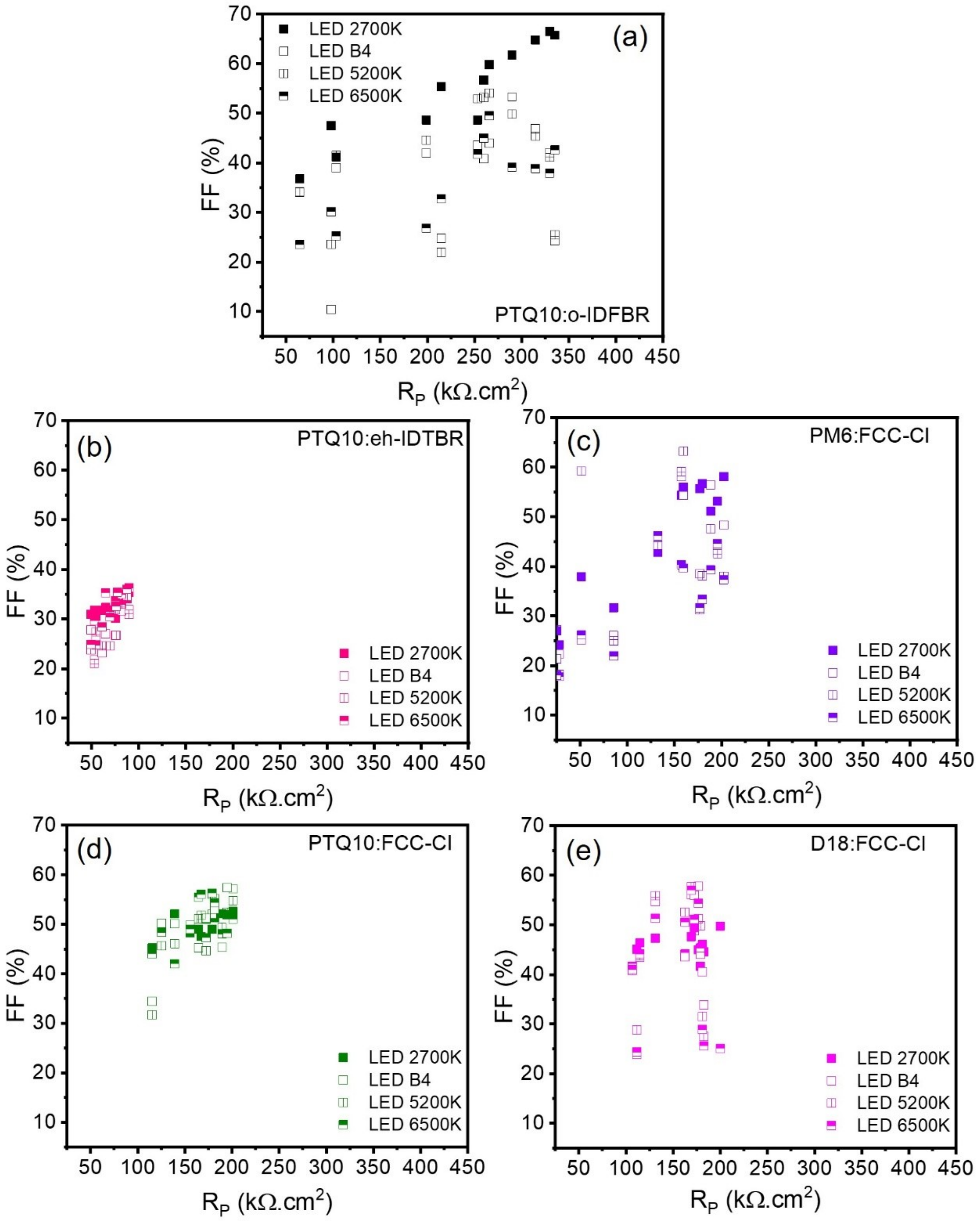

Figure S9. Indoor FF is plotted against R_P under all indoor light sources for (a) PTQ10:o-IDFBR, (b) PTQ10:eh-IDTBR, (c) PM6:FCC-Cl, (d) PTQ10:FCC-Cl, and (e) D18:FCC-Cl.

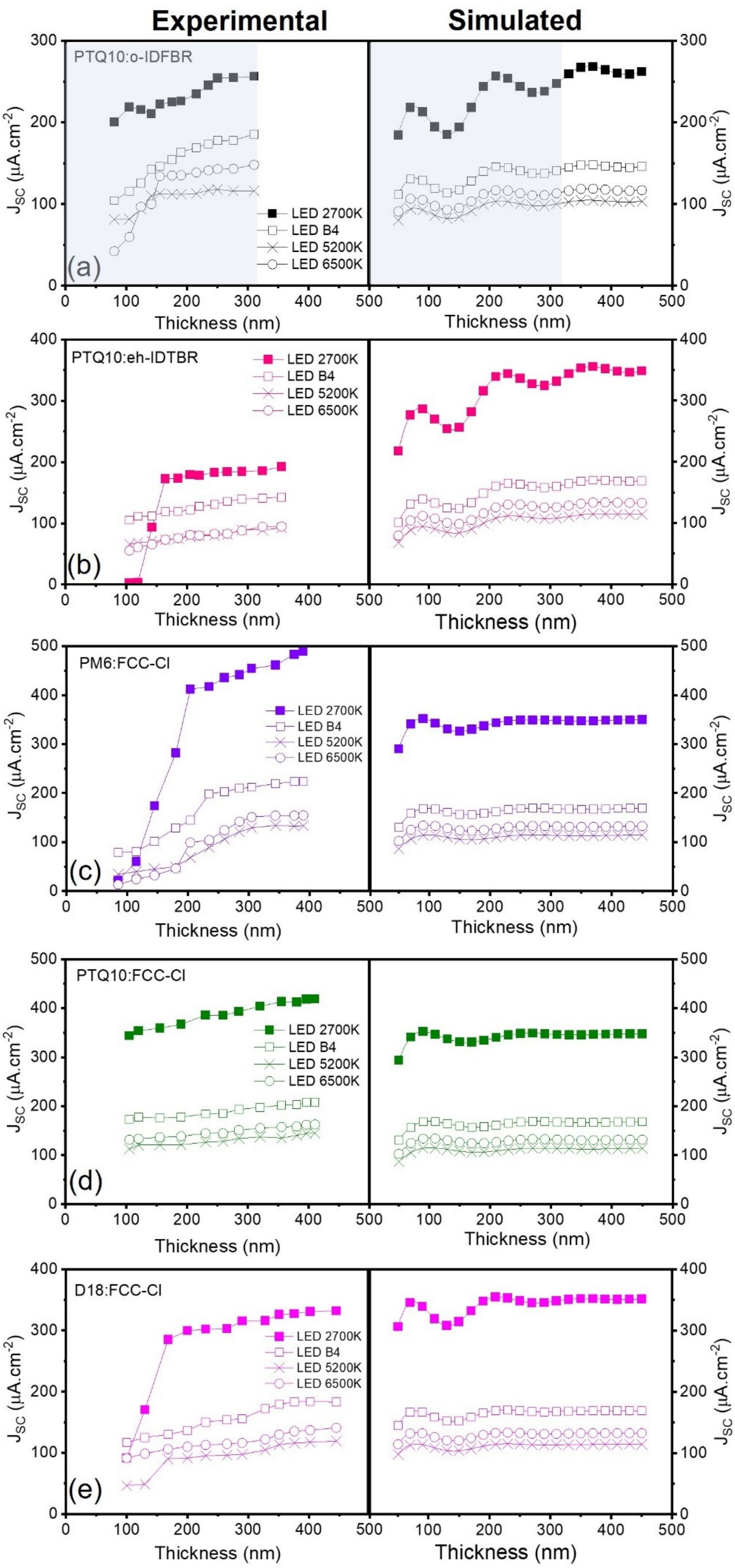

Experimental
Simulated
PTQ10:o-IDFBR
LED 2700K
LED B4
LED 5200K
LED 6500K
(a)
PTQ10:eh-IDTBR
(b)
PM6:FCC-Cl
(c)
PTQ10:FCC-Cl
(d)
D18:FCC-Cl
(e)
Thickness (nm)
J_{SC} (μA.cm^{-2})

Figure S10. Indoor J_{SC} (experimental vs simulated) is plotted against thickness gradient under all indoor light sources for (a) PTQ10:o-IDFBR, (b) PTQ10:eh-IDTBR, (c) PM6:FCC-Cl, (d) PTQ10:FCC-Cl, and (e) D18:FCC-Cl.

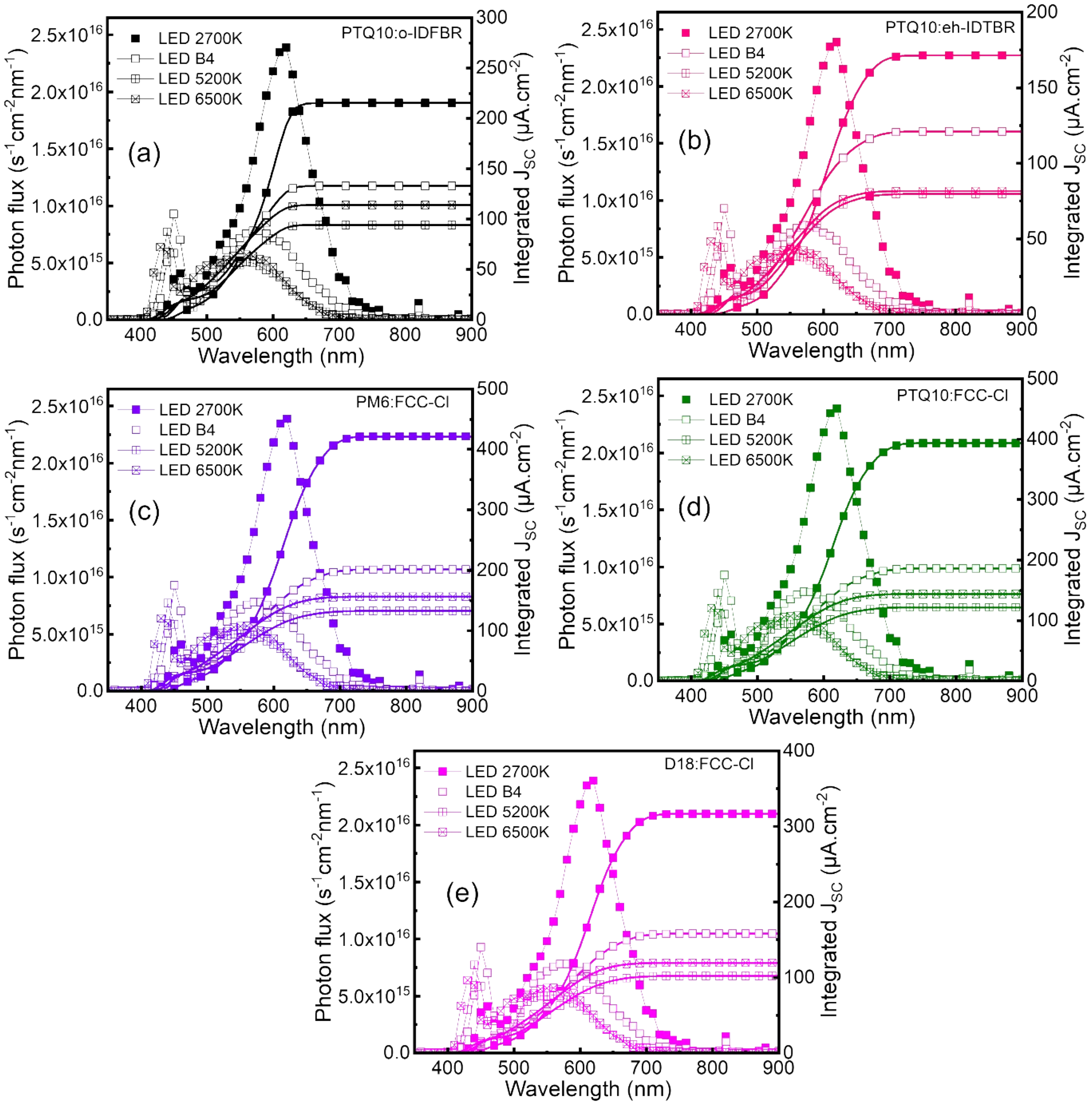

Figure S11. The photon flux and integrated J_{SC} (J_{EQE}: incorporating EQE and irradiation spectra of 2700K LED) of best performing cells under four indoor light sources for (a) PTQ10:o-IDFBR, (b) PTQ10:eh-IDTBR, (c) PM6:FCC-Cl, (d) PTQ10:FCC-Cl, and (e) D18:FCC-Cl.

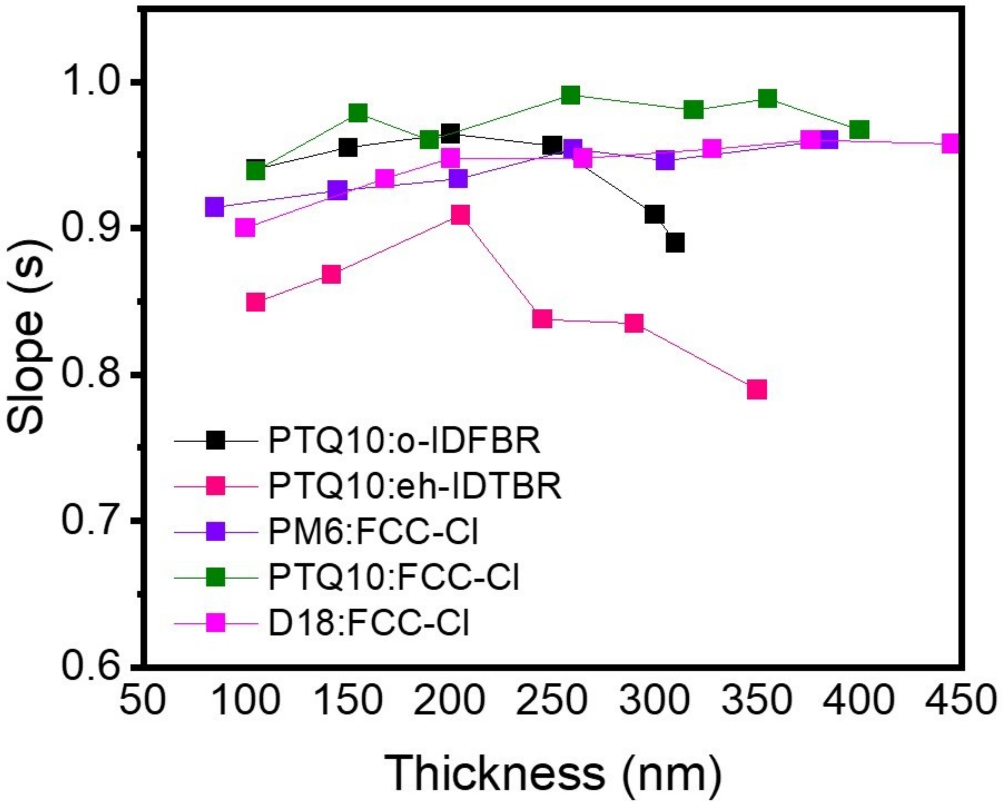

Figure S12. Thickness dependent slope values for all active layers, calculated through by varying light intensity of 2700K LED.

Table S2. A summary of state-of-the-art indoor OPVs under diverse indoor illumination LED conditions.

Active layer materials	LED	Irradiance ($\mu W.cm^{-2}$)	Intensity (lux)	V_{OC} (V)	J_{SC} ($\mu A.cm^{-2}$)	FF (%)	PCE (%)	Ref.
D18: Cl-BTA5	2700K	152	500	0.988	43.8	67.7	19.4	1
D18: Cl-BTA5	2700K	307	1000	1.01	91.1	68.9	21.1	1
D18: Cl-BTA5	6500K	307	1000	1.017	90.48	69.39	19.77	1
PBDB-T:F-M	2700K	152	216	0.740	35.6	64.2	11.2	2
D18:FCC-Cl	2600K	637	2000	0.975	245	80.1	30.1	3
PM6:FCC-Cl	2600K	637	2000	0.914	244	81.2	28.5	3
PM6:FCC-Cl	2600K	318	1000	0.895	122	81.1	27.9	3
PBDB-TF:Y6	2600K	--	20,000	0.812	2540	78.4	25.6	4
PB2:FCC-Cl	2600K	--	20000	1.02	2530	80.5	33.0	4
PTB7-Th:PC71BM	2600K	--	20000	0.734	2060	66.7	16.0	4
PB2:FCC-Cl	2700K	310	1000	0.926	120	80.7	30.4	5
PB4:FTCC-Br	2700K	305	1000	0.967	119	82.0	31.0	6
PM6:IO-4Cl	6500K	--	500	0.987	38.3	63.3	16.8	7
PPDT2FBT:PC70BM	2700K	280	1000	0.587	117	65.2	16.0	8
PTQ10:o-IDFBR*	12200K	200	560	1.21	57.7	58.5	22.6	9
PM6:ITIC-4F	White LED	332	1000	0.67	112.8	61.7	14.1	10
PM6:ITIC-4F	White LED	72	200	0.62	27.4	62.6	14.8	10
PTQ-10:ITIC-4F	White LED	332	1000	0.71	96	55.5	11.6	10
PTQ-10:ITIC-4F	White LED	72	200	0.63	24.4	51.2	11.3	10
PM6:L8-BO	3000K	254	1000	0.678	127	75.5	25.5	11
PBDB-T:N2200:PS	2700K	--	1200	0.73	101	76.1	15.7	12
P3TEA:FTTB-PDI4	3000K	--	1650	1.01	179	66.8	24.2	13
PM6/F-BTA3*	3000K	--	4000	1.1	2981	72.5	20.0	14
PM6:IT4F:ITIC-Th	3000K	160	500	0.750	73.1	77.1	29.1	15
PM6:IT4F:ITIC-Th	6500K	300	1000	0.74	19.9	70.1	22.4	15
P1:PCBM	3000K	77.6	300	0.758	29.5	66.1	19.1	16
PTQ10:o-IDFBR*	2700K	952	2800	0.743	222	64.8	11.2	This work
PTQ10:eh-IDTBR*	2700K	952	2800	0.987	178	35.4	6.55	
PM6:FCC-Cl*	2700K	952	2800	0.939	461	58.1	26.4	
PTQ10:FCC-Cl*	2700K	952	2800	0.953	414	52.6	21.7	
D18:FCC-Cl*	2700K	952	2800	0.969	327	49.4	16.5	
PTQ10:o-IDFBR*	B4	494	1700	0.733	154	48.3	11.2	
PTQ10:eh-IDTBR*	B4	494	1700	0.831	131	34.6	7.6	

PM6:FCC-Cl*	B4	494	1700	0.906	210	58.1	22.3
PTQ10:FCC-Cl*	B4	494	1700	0.940	202	57.1	22.0
D18:FCC-Cl*	B4	494	1700	0.984	179	57.8	20.6
PTQ10:o-IDFBR*	5200K	364	1200	0.769	113	53.2	12.69
PTQ10:eh-IDTBR*	5200K	364	1200	0.831	80.1	34.6	6.32
PM6:FCC-Cl*	5200K	364	1200	0.880	129	63.3	19.8
PTQ10:FCC-Cl*	5200K	364	1200	0.936	134	56.1	19.3
D18:FCC-Cl*	5200K	364	1200	0.961	104	57.7	15.9
PTQ10:o-IDFBR*	6500K	411	1400	0.769	135	39.1	9.9
PTQ10:eh-IDTBR*	6500K	411	1400	0.856	82.9	36.04	6.22
PM6:FCC-Cl*	6500K	411	1400	0.804	150	39.7	11.7
PTQ10:FCC-Cl*	6500K	411	1400	0.939	151	56.3	19.4
D18:FCC-Cl*	6500K	411	1400	0.962	122	57.0	16.3

*blade-coated active layers

References

1 Z. Wang, A. Tang, H. Wang, Q. Guo, Q. Guo, X. Sun, Z. Xiao, L. Ding and E. Zhou, *Chem. Eng. J.*, 2023, **451**, 139080.

2 D. Lübke, P. Hartnagel, M. Hülsbeck and T. Kirchartz, *ACS Mater. Au*, 2023, **3**, 215–230.

3 F. Bai, J. Zhang, A. Zeng, H. Zhao, K. Duan, H. Yu, K. Cheng, G. Chai, Y. Chen, J. Liang, W. Ma and H. Yan, *Joule*, 2021, **5**, 1231–1245.

4 W. Wang, Y. Cui, T. Zhang, P. Bi, J. Wang, S. Yang, J. Wang, S. Zhang and J. Hou, *Joule*, 2023, **7**, 1067–1079.

5 W. Wang, Y. Cui, Y. Yu, J. Wang, C. Wang, H. Hou, Q. Kang, H. Wang, S. Chen, S. Zhang, H. Xia and J. Hou, *Nano Energy*, 2024, **128**, 109893.

6 P. Bi, C. An, T. Zhang, Z. Chen, Y. Xu, Y. Cui, J. Wang, J. Li, Y. Wang, J. Ren, X. Hao, S. Zhang and J. Hou, *J. Mater. Chem. A*, 2022, **11**, 983–991.

7 D. Müller, L. Campos Guzmán, E. Jiang, B. Zimmermann and U. Würfel, *Sol. RRL*, 2022, **6**, 2200175.

8 S. C. Shin, C. W. Koh, P. Vincent, J. S. Goo, J. H. Bae, J. J. Lee, C. Shin, H. Kim, H. Y. Woo and J. W. Shim, *Nano Energy*, 2019, **58**, 466–475.

9 M. Casademont-Viñas, D. Capolat, A. Quesada-Ramírez, M. Reinfelds, G. Trimmel,

M. Sanviti, J. Martín, A. R. Goñi, T. Kirchartz and M. Campoy-Quiles, *J. Mater. Chem. A*, 2024, **12**, 16716–16728.

10 P. Perkhun, A. Khodr, Y. Alejandra, A. Quiroz, A. Karahan, H. Alkhatib, A. K. Bharwal, D. Duché, J. Simon, C. M. R. Herrero, T. Watanabe, H. Sekimoto, N. Yoshimoto, O. Margeat and C. Videlot-ackermann, *Energies*, 2026, **19**, 1773.

11 S. Oh, Y. Kang, T. H. Kim, S. J. Kim, M. J. Lee, G. M. Lee, M. A. Saeed and J. W. Shim, *JPhys Energy*, 2024, **6**, 025015.

12 T. Wang, Z. C. Wen, L. H. Xu, C. C. Qin, H. Yin, J. Q. Liu and X. T. Hao, *J. Mater. Chem. A*, 2021, **9**, 13515–13521.

13 H. Yin, L. K. Ma, J. Yan, Z. Zhang, A. M. H. Cheung, J. Zhang, H. Yan and S. K. So, *Sol. RRL*, 2020, **4**, 2000291.

14 J. Gao, Z. Ma, F. Zhao, Z. Li, Y. Lin, M. Wang, D. Ge and Z. Tang, *Small*, 2025, **21**, e06344.

15 C. Lee, J. H. Lee, H. H. Lee, M. Nam and D. H. Ko, *Adv. Energy Mater.*, 2022, **12**, 2200275.

16 H. Yin, S. Chen, S. H. Cheung, H. W. Li, Y. Xie, S. W. Tsang, X. Zhu and S. K. So, *J. Mater. Chem. C*, 2018, **6**, 9111–9118.
